# Targeting miR-181a/b in retinitis pigmentosa: implications for disease progression and therapy

**DOI:** 10.1186/s13578-024-01243-3

**Published:** 2024-05-21

**Authors:** Bruna Lopes da Costa, Peter M. J. Quinn, Wen-Hsuan Wu, Siyuan Liu, Nicholas D. Nolan, Aykut Demirkol, Yi-Ting Tsai, Salvatore Marco Caruso, Thiago Cabral, Nan-Kai Wang, Stephen H. Tsang

**Affiliations:** 1 Jonas Children’s Vision Care (JCVC) and Barbara & Donald Jonas Stem Cell Laboratory, New York-Presbyterian Hospital, New York, NY USA; 2 Department of Biomedical Engineering, Columbia University, New York, NY USA; 3 Department of Ophthalmology, Columbia University Irving Medical Center, New York, NY USA; 4 Department of Specialized Medicine, CCS and Vision Center Unit, Ophthalmology EBSERH, HUCAM/CCS, UFES-Federal University of Espírito Santo (UFES), Vitória, Brazil; 5 Department of Ophthalmology, Federal University of Sao Paulo (UNIFESP), São Paulo, Brazil; 6 Department of Pathology & Cell Biology, Columbia University Irving Medical Center, New York, NY USA; 7 Columbia Stem Cell Initiative, Institute of Human Nutrition ,Vagelos College of Physicians and Surgeons, Columbia University Irving Medical Center, New York, NY USA; 8 Columbia University Irving Medical Center, Hammer Health Sciences Center 205b, 701 West 168th Street, New York, NY 10032 USA

**Keywords:** MicroRNAs, Metabolic reprogramming, Retinal pigment epithelium, Retinitis pigmentosa, Aerobic glycolysis

## Abstract

**Background:**

Retinitis pigmentosa (RP) is a genetically heterogeneous group of degenerative disorders causing progressive vision loss due to photoreceptor death. RP affects other retinal cells, including the retinal pigment epithelium (RPE). MicroRNAs (miRs) are implicated in RP pathogenesis, and downregulating miR-181a/b has shown therapeutic benefit in RP mouse models by improving mitochondrial function. This study investigates the expression profile of miR-181a/b in RPE cells and the neural retina during RP disease progression. We also evaluate how miR-181a/b downregulation, by knocking out miR-181a/b-1 cluster in RPE cells, confers therapeutic efficacy in an RP mouse model and explore the mechanisms underlying this process.

**Results:**

Our findings reveal distinct expression profiles, with downregulated miR-181a/b in RPE cells suggesting a protective response and upregulated miR-181a/b in the neural retina indicating a role in disease progression. We found that miR-181a/b-2, encoded in a separate genomic cluster, compensates for miR-181a/b-1 ablation in RPE cells at late time points. The transient downregulation of miR-181a/b in RPE cells at post-natal week 6 (PW6) led to improved RPE morphology, retarded photoreceptor degeneration and decreased RPE aerobic glycolysis.

**Conclusions:**

Our study elucidates the underlying mechanisms associated with the therapeutic modulation of miR-181a/b, providing insights into the metabolic processes linked to its RPE-specific downregulation. Our data further highlights the impact of compensatory regulation between miR clusters with implications for the development of miR-based therapeutics.

**Graphical Abstract:**

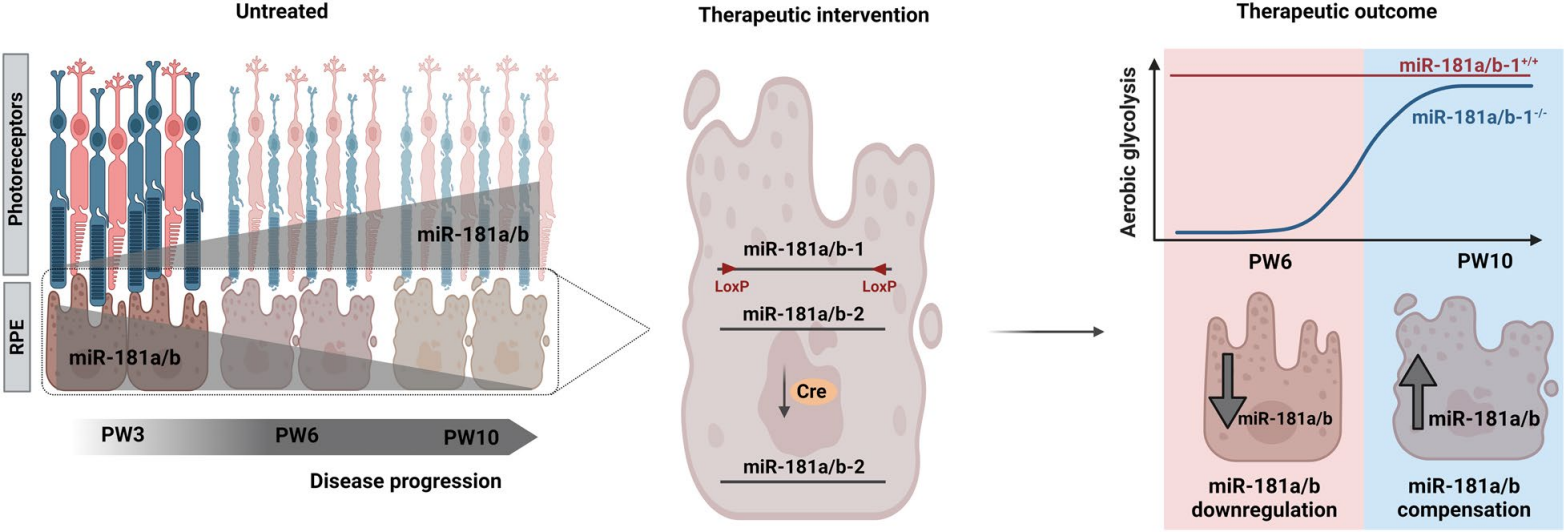

**Supplementary Information:**

The online version contains supplementary material available at 10.1186/s13578-024-01243-3.

## Introduction

Retinitis pigmentosa (RP) is a genetically heterogeneous group of retinal disorders causing progressive vision loss due to photoreceptor (PR) degeneration, ultimately leading to blindness. While rod PRs are primarily affected in RP, their death triggers secondary processes that result in the demise of cone PRs (1). Nevertheless, the progressive alterations observed in the RP retina extend beyond the PRs, impacting other cell types within the retina, such as the retinal pigment epithelium (RPE) [2–4]. The RPE cells provide essential support for retinal function by transporting nutrients and maintaining the outer segments of PRs [5, 6]. RP pathology can adversely affect RPE function, exacerbating disease progression.

The genetic heterogeneity of RP, with over 60 identified associated genes [7], poses challenges in understanding disease mechanisms and developing effective treatments for RP. However, several factors, including mitochondrial dysfunction, oxidative stress, glycolytic dysregulation, and microglia activation, have been implicated in RP pathogenesis and progression [8–10]. Hence, identifying and targeting shared pathways affected in RP holds promise for therapeutic interventions that can benefit patients irrespective of their underlying genetic defects [11]. In this scenario, microRNAs (miRs) are considered as potential therapeutic targets. These small non-coding molecules can post-transcriptionally regulate gene expression and modulate various biological processes in health and disease [12, 13]. Dysregulation of miRs has been observed in retinal disorder, which may represent compensatory survival mechanisms or contribute to disease progression [14–17]. Importantly, miR modulation has demonstrated beneficial effects for RP treatment [18–20].

Recently, miR-181a/b was found to be highly expressed in the human retina [21], and it has been shown to participate in the reprogramming of cancer metabolism [22–24]. In particular, the downregulation of miR-181b-5p suppressed glycolysis of gallbladder cancer by upregulating PDHX, decreasing the viability of the cancer cells [23]. Furthermore, overexpression of miR-181a promotes the proliferation of colon cancer cells through activation of the PTEN/AKT pathway, which enhances glycolysis in the tumor cells [24]. Similarly to PRs, cancer cells use glucose to produce energy and lactate through aerobic glycolysis even when oxygen is available-a process known as Warburg aerobic glycolysis [25, 26]. Conversely, RPE cells depend on mitochondrial oxidative phosphorylation (OXPHOS) and reductive carboxylation for energy production [27, 28]. Importantly, mitochondrial dysfunction of RPE cells leads to an increased reliance on glycolysis [8, 29]. Consequently, less glucose is transported from the RPE to the retina, contributing to the death of PR cells [1, 3, 30]. Therefore, reprogramming retinal metabolism to restore the metabolic coupling between PRs and RPE cells has been proposed as a promising therapeutic strategy for retinal disorders [31]. Specifically, enhancing aerobic glycolysis in rod PRs [32] and increasing glucose uptake by PRs have been shown to preserve cell survival in various RP mouse models [33–35].

The downregulation of miR-181a/b has shown therapeutic benefits in neurodegenerative disorders, including RP caused by mutations in the *Rhodopsin* (*Rho*) and *Phosphodiesterase 6β* (*Pde6β*) genes [18, 36]. The use of an adeno-associated viral vector (AAV) carrying a miR-181a/b complementary sequence, referred to as a “sponge”, improved mitochondrial morphology and function in PR cells. Notably, using a ubiquitous promoter (PRs and RPE transduction) resulted in a more significant rescue effect than cell-type-specific expression of the miR-181a/b “sponge” in rods or RPE cells alone. This data suggests that simultaneous downregulation of miR-181a/b in multiple cell types within the retina is crucial for achieving optimal therapeutic outcomes. Surprisingly, no therapeutic benefits were observed when an RPE-specific promoter was utilized [18]. However, in vivo delivery of episomal vectors to the mouse RPE using a ubiquitous promoter such as cytomegalovirus (CMV) resulted in higher transgene expression than RPE-specific promoters [37]. The gene expression level directly influences the detectability of therapeutic outcomes, and when expression levels are low, beneficial effects that could have been observed may remain hidden. Despite extensive efforts to investigate the therapeutic potential of downregulating miR-181a/b in the treatment of retinal disorders, the expression patterns of miR-181a/b in the neural retina (NR) and RPE during the progression of RP have not been explored. RP affects both PR cells and RPE cells, and it is possible that the dysregulation of miR-181a/b expression differs between these cell types.

In this study, to investigate these aspects, we examined the changes in miR-181a/b expression during RP progression in both RPE cells and NR. Furthermore, we proposed and tested the hypothesis that, apart from improving mitochondrial function, downregulating miR-181a/b in RPE cells may also regulate the metabolic state of these cells. Our findings revealed distinct changes in miR-181a/b levels during disease progression in RPE cells and NR compared to the wild-type (WT). Notably, miR-181a/b decreased in RPE cells, indicating a response to combat the disease, while miR-181a/b increased in the NR, suggesting its involvement in disease progression. The downregulation of miR-181a/b in RPE cells was associated with decreased aerobic glycolysis, as evidenced by the downregulated expression of lactate dehydrogenase A (LDHA). The downregulation of miR-181a/b was linked to an improved phenotype in the *Pde6β*^*H620Q*^ RP mouse model. Through our study, we offer insights into the therapeutic potential of metabolic regulation associated with miR-181a/b downregulation in RPE cells and demonstrate compensatory mechanisms that might hold significant impact for developing miR-based therapies in retinal diseases.

## Methods

### Animals

All mice were housed in the Columbia University Pathogen-Free Eye Institute Animal Facility and maintained with a 12 h light/12 h dark cycle with free access to food and water. We crossed three lines of mice to develop the breeding strains. Mirc14^tm1.1Czc^ mice [38] were purchased from the Jackson Laboratory; *Pde6β*^*H620Q*^ mice were rederived via oviduct transfer using European Mouse Mutant Archive (EMMA) morulae [39]; and *Rpe65*^*CreERT2*^ mice were generated in the Barbara & Donald Jonas Stem Cell and Regenerative Medicine Laboratory. *Pde6β*^*H620Q*^ mice were crossed with *Rpe65*^*CreERT2*^ mice, and their offspring were bred with Mirc14^tm1.1Czc^ mice. At least six generations of backcrosses were required to generate the mice with the desired genotype. The resulting progeny were homozygous for *Pde6β*^*H620Q*^ and miR-181a/b-1^ fl/fl^ and heterozygous or wild-type for *Rpe65*^*CreERT2*^. These two lines are the breeding strains and those mice were never exposed to tamoxifen injection. The crossing of the breeding strains produced the experimental mice, which 50% are expected to be homozygous for *Pde6β*^*H620Q*^ and miR-181a/b-1^ fl/fl^ and heterozygous for *Rpe65*^*CreERT2*^ (treated group, Pde6β^H620Q^miR-181a/b-1^−/−^) and the remaining 50% have a similar genotype but wild-type for *Rpe65*^*CreERT2*^ (control group, Pde6β^H620Q^miR-181a/b-1^+/+^). Similarly, homozygous mice for *Pde6β*^*WT*^ and miR-181a/b-1^ fl/fl^ and heterozygous or wild-type for *Rpe65*^*CreERT2*^ were crossed to generate, Pde6β^WT^miR-181a/b-1^−/−^ (WT treated) and Pde6β^WT^miR-181a/b-1^+/+^ (WT control). *Pde6β*^*WT*^ mice have been selectively bred to be congenic with *Pde6β*^*H620Q*^ mice, ensuring a valid comparison between the control and experimental groups.

### Tamoxifen injection and sample collection

Tamoxifen (100 mg/ml in ethanol; Sigma-Aldrich) was diluted with sunflower seed oil (Sigma-Aldrich) to a concentration of 10 mg/ml and thoroughly mixed at 42 °C. Two intraperitoneal injections (dose of 0.1 mg/g) were administered between P8-P12 in both treated and control mice. There was no discrimination based on the sex of the mice. Animals were euthanized by CO_2_ followed by cervical dislocation at three time points: 3, 6, and 10 weeks after birth. Samples were collected for the downstream experiments, and confirmation of miR-181a/b-1 ablation was evaluated in every single mouse. The absence of a predominant excised band, indicating an inefficient gene ablation was the the criterion for mice exclusion. Genetic validation was essential to confirm that subsequent analyses, encompassing PRs function and morphology, along with assays involving RNA and protein samples, were conducted solely in mice exhibiting predominant miR-181a/b-1 ablation in RPE cells.

### RNA extractions

Total RNA from eye cup or neural retina was extracted using the RNeasy Extraction Kit (Qiagen), according to the manufacturer’s instructions. Eye cups or neural retinas were placed in the buffer RLT for homogenization and RNA extraction, and the remaining tissues were placed in Dulbecco’s phosphate-buffered saline (DPBS) for subsequent genomic DNA extraction. Homogenization of eye cups was conducted gently to ensure the enrichment of RPE cells.

### Genomic DNA extraction and PCR

Genomic DNA was extracted as previously described [40]. In brief, eye cups or neural retina were placed in DPBS and gently homogenized. Homogenization of eye cups was conducted gently to ensure the enrichment of RPE cells. Subsequently, the remaining eye cups were removed for the subsequent DNA extraction of the RPE cells collected in the supernatant.The resultant solution was incubated for 20 min at 95 °C. Proteinase K (Qiagen) was added, followed by 1 h incubation at 56 °C and 30 min at 95 °C. Genomic PCRs were performed using Phire DNA polymerase (Fisher Scientific). Primers for the confirmation of the ablation of the target gene were: forward-5′ TAACTTGAGAAAACCTAAGTGG 3′ and reverse–5′ TCCCTGGAATCAAGTCTAC 3′ [38]. Excised bands (306 bp) were further subcloned by the TOPO Blunt-End kit (Invitrogen) and analyzed by Sanger sequencing.

### Mature miRNA quantitative assay

Mature miR-181a and miR-181b were quantitatively detected using specific miRs probes (TaqMan MicroRNA assays, Thermo Fisher Scientific). cDNAs for mature miR-181a/b analysis were generated with miR-specific primers using the TaqMan MicroRNA Reverse Transcription Kit, following the manufacturer’s instructions. The quantification data was obtained using TaqMan^™^ Fast Advanced Master Mix (Thermo Fisher Scientific) on TaqMan-cDNAs from control and treated groups were represented in terms of cycle thresholds (Ct). The RNA sno202 TaqMan probe served as the endogenous control for the experiments, chosen for its abundant and stable expression across various tissues [41]. Ct values were analyzed as described in the “Quantitative Real-Time PCR” section. Each reaction was done in technical duplicate, and the results are presented as means ± SEM of at least three independent biological replicates.

### Quantitative real-time PCR

RNA was collected as described in the “RNA extractions” section. DNase I treatment (Invitrogen) was performed to prevent genomic DNA contamination. The reverse transcription reaction utilized the Superscript III reverse transcription kit, with an oligo(dT)20 (Invitrogen) for cDNA synthesis. Real-time PCR was carried out using the SsoAdvanced Universal SYBR Green Supermix (Bio-Rad) and the CFX Connect real-time PCR detection system (Bio-Rad) to quantify gene expression levels. To normalize the data, endogenous β-actin mRNA expression levels were determined. The primer sequences used for qPCR can be found in Table S1. The Ct values were averaged for each in-plate technical duplicate, and the averaged Ct was then normalized as the difference in Ct values (ΔCt) between the analyzed mRNAs and each reference gene in each sample analyzed. The variation was reported as a relative fold change (2^(−ΔCt)^). The results are presented as means ± SEM of at least three independent biological replicates.

### Quantitative mitochondrial DNA content analysis

We conducted SYBR Green qPCR using primers targeting the MT-CO1 gene (mtDNA) and the RNaseP gene (nuclear reference gene), following the methods described previously [36, 42]. The mtDNA relative copy number was calculated based on the threshold cycle value (ΔCt), and the mtDNA copy number per cell was determined as 2 × 2^(−ΔCt)^ to account for the presence of two copies of RNaseP in each nucleus. The results are presented as means ± SEM of at least three independent biological replicates.

### Protein isolation and immunoblots

Neural retina and eye cups were collected at specified time points and directly lysed in 100µL of M-PER Mammalian Protein Extraction Reagent (Thermo Scientific), supplemented with protease inhibitors (Sigma-Aldrich), while maintaining constant agitation at 4 °C for 30 min. After sonication, the lysates were centrifuged at 20,000 × g and 4 °C for 10 min, and the resulting supernatant was collected for gel loading. Subsequently, 30 µL aliquots of the lysates were mixed with 4 × Laemmli sample buffer (Bio-Rad) and incubated at 95 °C for 10 min. The samples were then separated using a 4%–15% Bis-Tris gel (Bio-Rad) and transferred onto nitrocellulose membranes (Bio-Rad). The membranes were blocked for 30 min in EveryBlot blocking buffer (Bio-Rad). Next, the membranes were incubated overnight at 4 °C with primary antibodies diluted in the blocking buffer. The primary antibodies used were rabbit anti-PKM2 polyclonal antibody (1:1000; Cell signaling, 3198), rabbit anti-pPKM2 (phosphorylation Tyr105) polyclonal antibody (1:1000; Cell signaling, 3827), rabbit anti-LDHA polyclonal antibody (1:1000; Thermo Fisher Scientific, PA5-23036), and mouse anti-β-actin monoclonal antibody (1:2000; Cell signaling, 8H10D10). After the overnight incubation, the membranes were washed three times with PBS containing 0.1% (v/v) Tween 20 (PBST) for 10 min each and then incubated with secondary antibodies for 2 h at room temperature. The secondary antibodies used were rabbit anti-mouse IgG-HRP antibody (1:2000; Abcam ab6728) and mouse anti-rabbit IgG-HRP antibody (1:2000; Abcam ab99697). Following another three washes with PBST for 10 min each, protein bands were manually visualized using the Med-Dent ready-to-use developer/fixer combo (Z&Z Medical) after exposure to Immobilon western chemiluminescent substrate (Millipore).

### Histology

Mice were euthanized, and the eyes were enucleated and fixed in Z-Fix zinc formalin fixative. Eyes were embedded in paraffin, and orientated retinal sections were obtained through the optic nerve. Hematoxylin and eosin (H&E) staining was conducted as previously described [43, 44]. Images were obtained using a Leica SCN400 whole-slide digital imaging system and analyzed using Aperio ImageScope (Leica). The length of ONL and PS was measured throughout the retina by two independent blind investigators. The final results are presented as the average of the measurements determined by the two observers for each biological replicate ± SEM.

### Electroretinography (ERG)

ERGs of Rpe65^+/+^, Rpe65^+/CreERT2^, and Rpe65^CreERT2^ mice were performed in anesthetized mice at 5 months-old as previously reported by our group [45]. ERGs of Pde6β^H620Q^miR-181a/b-1^+/+^ and Pde6β^H620Q^miR-181a/b-1^−/−^ mice were performed in anesthetized 6 weeks and 10 weeks old mice as previously described [32]. In brief, Espion V6 Diagnosys ColorDome (Diagnosys) was used to record ERG responses concurrently from both eyes. For rod and maximal rod and cone ERG responses, pulses of 0.001 cd/m^2^ and 3 cd/m^2^ (White-6500 K) were used, respectively. Each result represents an average of 40–60 trials. For cone responses, mice were light-adapted in the Ganzfeld dome for 10 min. A background of 30 cd/m^2^ (White-6500 K) was present throughout the trials to suppress rod function. ERGs were recorded using white flashes. The responses recorded simultaneously from both eyes were averaged as data to represent each mouse for further analysis. The number of mice per group is described in the figure legends.

### Immunofluorescence analysis

For immunofluorescence analysis, mouse eyes were first fixed in Z-Fix and then embedded in paraffin. To prepare the tissue for staining, deparaffinization was carried out by immersing the slides in a series of solutions for three minutes each: (1) xylene, (2) xylene, (3) a mixture of xylene and 100% ethanol in a 1:1 ratio, (4) 100% ethanol, (5) 95% ethanol, (6) 70% ethanol, (7) 50% ethanol, and finally (8) water. The slides were boiled at 100 ℃ in Citrate Buffer (pH 6.0, 10 × concentration from Sigma-Aldrich) for 20 min for antigen retrieval. The slides were allowed to cool down to room temperature after the boiling step. Next, the prepared slides were incubated overnight at 4 ℃ with the primary antibodies: anti-Rhodopsin (Abcam, ab5417, 1:50), anti-C-Arrestin (Millipore AB15282, 1:50). To visualize the antigen–antibody complexes, fluorochrome-labeled secondary antibodies (Alexa Fluor, diluted 1:1000; Invitrogen) were used, and the cell nuclei were labeled with DAPI (Millipore Sigma). Finally, the sections were imaged using a DM5000B Leica fluorescence microscope to capture the immunofluorescence signals. Images were taken from each section at the central retina, capturing comparable regions equidistant from the optic nerve. For the quantification of rhodopsin internalization into the cells’ cytoplasm the fluorescent images were converted to grayscale. Fluorescence signal intensity was quantified for each region of interest as integrated density (IntDen) after background subtraction using ImageJ. The quantification of cone pedicles was carried out manually.

### RPE morphometric analysis

RPE–choroid–sclera flat mounts were stained for phalloidin (Abcam, ab176756, 1:1000). Confocal imaging of the central region of the flat mount was acquired using an AXR resonant scanning confocal attachment on a Ti2 microscope (Nikon Instruments) using a 40x/0.75 NA Plan Apo VC objective lens. The pinhole was set to 1 Airy Unit. Tiled Z-stacks were collected using sequential scanning with standard lasers and emission bandpasses. NIS Elements software (Nikon) was used to denoise images (Denoise.AI algorithm), and stitch the tiles together. Three separate biological replicates were included in each group examined. Six to eight equally sized regions of the central area were examined in each flat-mount. RPE cell area was quantified manually utilizing the ImageJ Software as previously reported [8]. Two independent observers, who were blinded to the experimental conditions, assessed the morphology of RPE cells qualitatively based on their size and shape. The scoring system ranged from 1 to 5, with a score of 1 indicating healthier morphology and 5 indicating diseased morphology. For each flat mount, the scores assigned by the two investigators were averaged. The results are presented as the mean of the average scores determined by the two observers for each biological replicate ± SEM. Schütze et al. [46] used a similar methodology to evaluate the morphologic parameters of the RPE and monitor atrophy progression in age-related macular degeneration (AMD) using polarization-sensitive optical coherence tomography (OCT) images.

### Statistical analysis

Statistical analyses were performed using GraphPad Prism software version 9.3.1. In all experiments, we ensured the inclusion of at least three animals per genotype to obtain statistically reliable results. For statistical analysis, Unpaired Student’s t-test and the Unpaired Student’s t-test with Welch’s correction were employed as appropriate, with the specific test indicated in each figure legend. One-way ANOVA compared ERG responses from Rpe65^+/+^, Rpe65^+/CreERT2^, Rpe65^CreERT2^ mice. Quantitative data are presented as the mean ± SEM (standard error of the mean) derived from at least three independent biological replicates, providing a robust representation of the results.

## Results

### miR-181a/b is dysregulated in the retina and RPE cells of the ***Pde6β***^***H620Q***^ RP mice

**Fig. 1:**
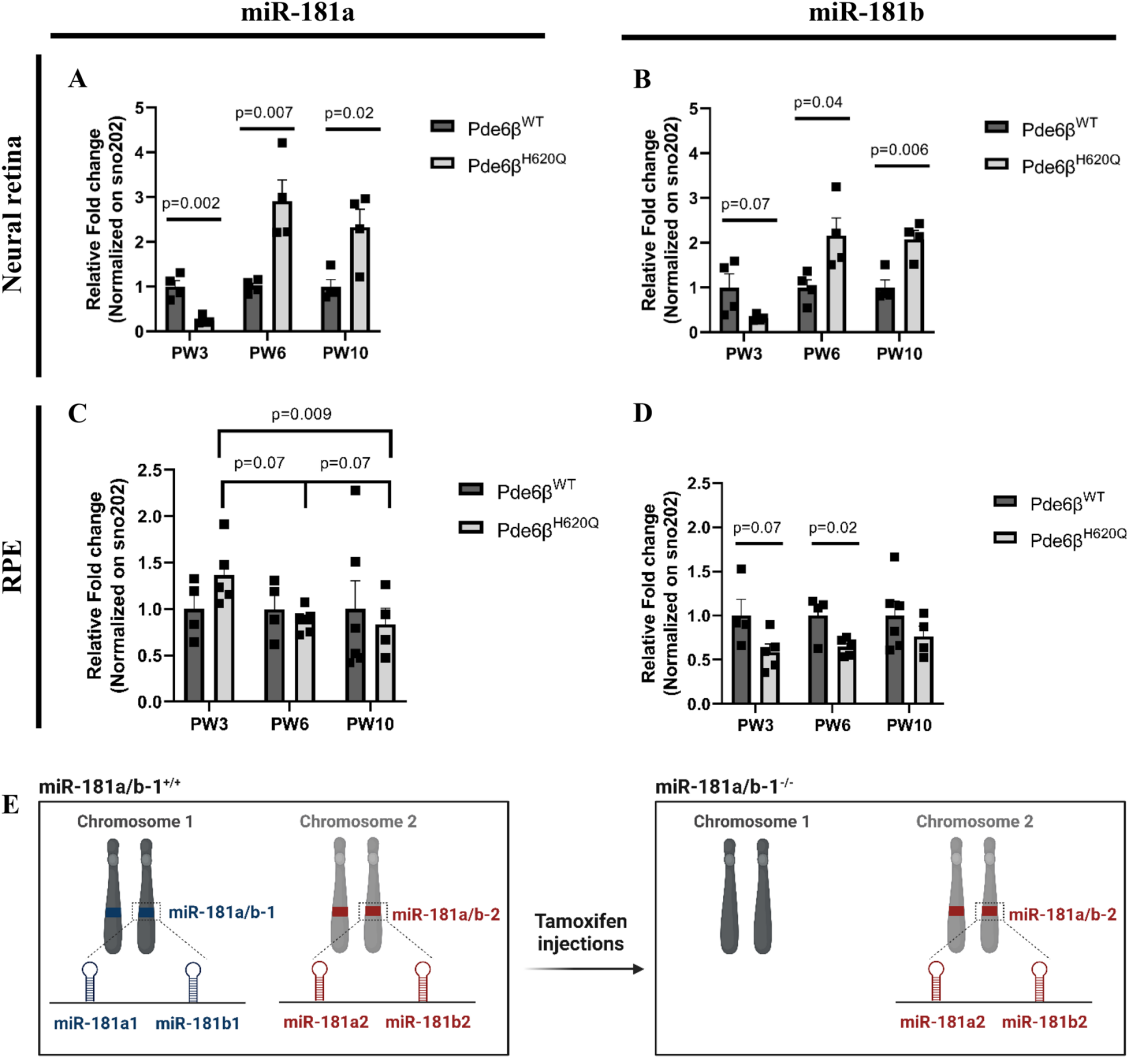
Expression of miR-181a and miR-181b are dysregulated in the NR and RPE of *Pde6β*^*H620Q*^ mice. **A**–**B** qPCR analysis of (**A**) miR-181a and **B** miR-181b expression in the NR of *Pde6β*^*WT*^ and *Pde6β*^*H620Q*^ mice at PW3, PW6, and PW10. N ≥ 4 mice. P-values are reported in black. Data are presented as mean ± SEM. Student’s t-test, unpaired. **C**–**D** qPCR analysis of (**C**) miR-181a and **D** miR-181b expression in the RPE cells of *Pde6β*^*WT*^ and *Pde6β*^*H620Q*^ mice at PW3, PW6, and PW10. N ≥ 4 mice. P-values are reported in black. Data are presented as mean ± SEM. Student’s t-test, unpaired. **E** Illustration of mouse model design. miR-181a/b are organized in two separate genomic clusters within mammalian cells: miR-181a/b-1 located on chromosome 1, and miR-181a/b-2 located on chromosome 2. Tamoxifen injection only mediates ablation of miR-181a/b-1

The *Pde6β*^*H620Q*^ mutant is a mouse model of autosomal recessive RP, which carries a homozygous missense mutation in the catalytic domain of the *Pde6β* gene. The resulting partial loss of cGMP hydrolysis causes a disease phenotype primarily affecting PR cells [39]. Notably, at post-natal week 3 (PW3), just before or at the initial stage of degeneration, miR-181a and miR-181b were downregulated in the NR of RP mice compared to WT controls. As the disease progressed (PW6 and PW10), the expression levels of miR-181a/b in the NR of RP mice increased significantly (Fig. 1A, B). Interestingly, in the NR of healthy WT mice, miR-181a and miR-181b expression levels remain stable throughout aging. In contrast, during disease progression in the *Pde6β*^*H620Q*^ mutant mice, the levels of miR-181a/b exhibited a statistically significant increase (Additional file 1: Fig. S1A–D), indicating their association with the degeneration of NR cells.

While PR cells are primarily affected in RP, secondary effects are known to impact RPE cells [2–4]. Interestingly, miR-181a expression in the RPE cells of the RP mice showed no significant changes compared to WT mice at the evaluated time points (Fig. 1C). However, miR-181b was downregulated in RPE cells of mice with retinal degeneration (Fig. 1D). It is worth noting that although the expression of miR-181a remained similar between the healthy and diseased states, its levels decreased over disease progression in the RP mice, which was not observed in the WT context (Fig. 1C, and Additional file 1: Fig. S1E, G). On the other hand, miR-181b exhibited a more stable expression throughout both health and disease progression in the RPE, but its downregulation began at the onset of degeneration (PW3) (Fig. 1D, and Additional file 1: Fig. S1F, H). These findings suggest that RPE cells respond to disease progression from early to late stages. Notably, while morphological remodeling of RPE cells became evident at PW16 [47], we observed molecular remodeling as early as PW3, characterized by lower levels of miR-181b in the disease and decreasing levels of miR-181a over disease progression. However, the precise nature of this molecular remodeling remains uncertain whether it represents a beneficial response against disease progression or contributes to the worsening of the disease phenotype. To address this question, we generated a mouse line that enables Cre-mediated downregulation of miR-181a/b specifically in RPE cells of *Pde6β*^*H620Q*^ or *Pde6β*^*WT*^ mice.

### miR-181a/b-2 compensates for the ablation of miR-181a/b-1 in the RPE cells of ***Pde6β***^***H620Q***^ mutant mice

The miR-181a/b family is highly conserved between species, including humans and rodents. These miRNAs are found in two separate genomic clusters within mammalian cells: miR-181a/b-1 (cluster 1) is located on chromosome 1, and miR-181a/b-2 (cluster 2) is located on chromosome 2. Despite being encoded in different genomic regions, miR-181a/b-1 and miR-181a/b-2 share identical mature sequences, which means they have the same “seed” sequence and common targets [48]. Earlier studies showed that double knockout mice for miR-181a/b-1 and miR-181a/b-2 had a decreased body size and lower survival rate, indicating that miR-181a/b plays a crucial role in regulating vital pathways in vivo [49]. Our study focuses on the ablation of miR-181a/b-1, which has previously been shown to rescue neurodegenerative disorders, including RP [18, 36]. Our model still expresses miR-181a/b-2, generating a downregulation of miR-181a/b instead of knockout (Fig. 1E).

Cre-loxP systems enable temporal control and tissue/cell-specific ablation of the gene of interest. The Mirc14^tm1.1Czc^ is a conditional ablatable strain in which the miR-181a/b-1 locus is flanked by loxP sites [38]. *Rpe65*^*CreERT2*^ mouse line expresses the Cre enzyme specifically in RPE cells upon administration of an estrogen modulator, such as tamoxifen. We crossed the Mirc14^tm1.1Czc^, *Rpe65*^*CreERT2*^, and *Pde6β*^*H620Q*^ mouse strains to obtain, in the same litters, Pde6β ^*H620Q*^*miR*-181a/b-1^+/+^ (control group) and *Pde6β*^*H620Q*^*miR*-181a/b-1^−/−^ (treated group) mice.

**Fig. 2:**
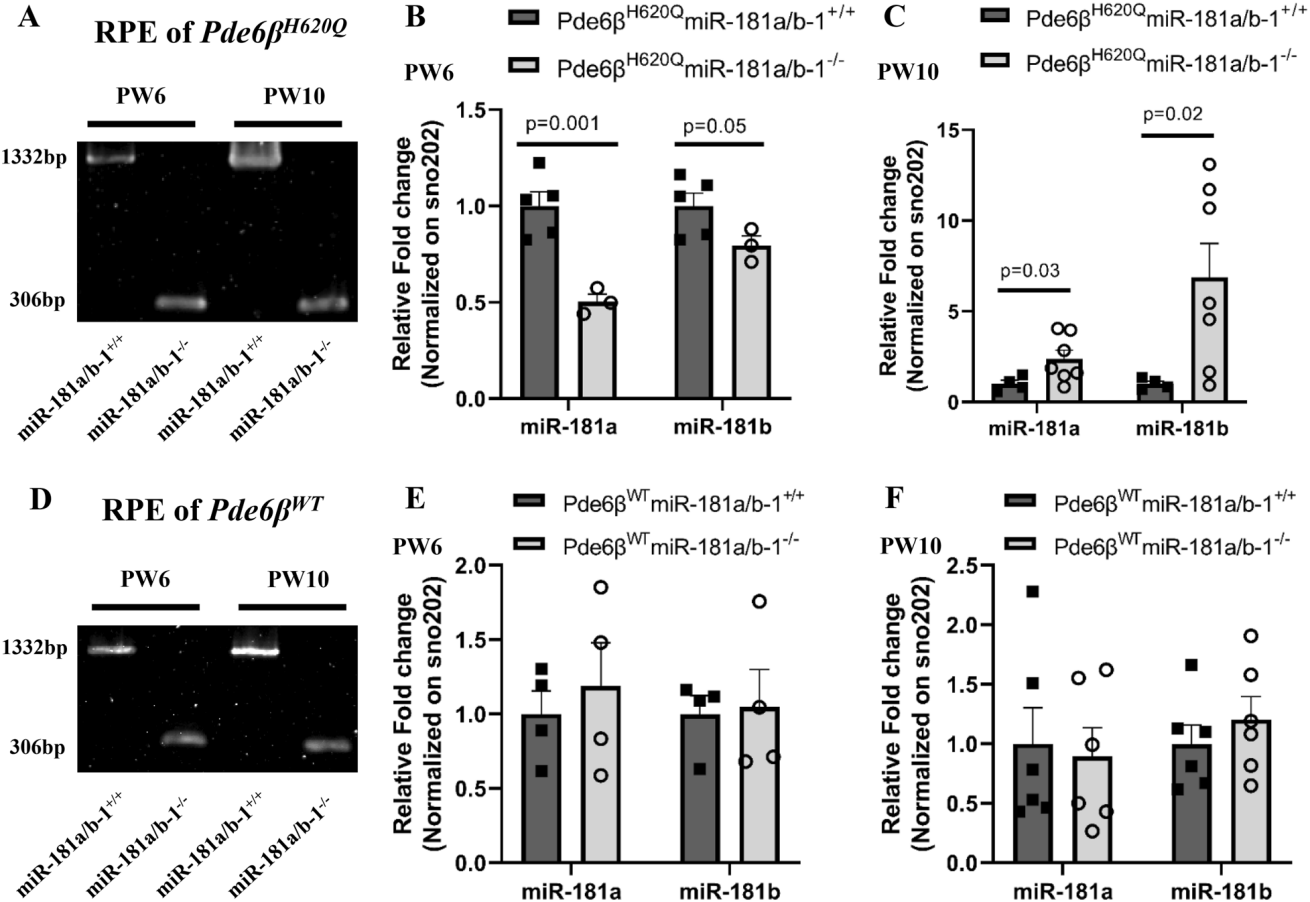
miR-181a/b shows dynamic expression upon tamoxifen injection in RPE cells of mutant mice. **A** Representative agarose gel electrophoresis result of genomic PCR to confirm miR-181a/b-1 ablation in the RPE cells of *Pde6β*^*H620Q*^ mice. **B**–**C** qPCR analysis of miR-181a and miR-181b expression in the RPE cells of Pde6β^H620Q^miR-181a/b-1^+/+^ and Pde6β^H620Q^miR-181a/b-1^–/−^ mice at (**B**) PW6, and **C** PW10. n ≥ 3 mice/genotype. P-values are reported in black. Data are presented as mean ± SEM. Student’s t-test, unpaired with Welch ‘s correction. **D** Representative agarose gel electrophoresis result of genomic PCR to confirm miR-181a/b-1 ablation in the RPE cells of *Pde6β*^*WT*^ mice. **E**–**F** qPCR analysis of miR-181a and miR-181b expression in the RPE cells of Pde6β^WT^miR-181a/b-1^+/+^ and Pde6β^WT^miR-181a/b-1^−/−^ mice at (**E**) PW6, and **F** PW10. n ≥ 4 mice/genotype. Data are presented as mean ± SEM. Student’s t-test, unpaired

We generated a new *Rpe65*^*CreERT2*^ Cre driver line with a genomic design resembling a previously published model [50]. The *Rpe65*^*CreERT/*+*2*^ mice showed normal retinal function and morphology, as assessed by ERG and histological sections, respectively (Additional file 2: Fig. S2A, B). Tamoxifen injected twice between post-natal day 8 (P8) and P12 successfully mediated the ablation of miR-181a/b-1, specifically in the RPE cells of *Pde6β*^*H620Q*^ mice, without miR-181a/b-1 excision in the NR (Fig. 2A, and Additional file 2: Fig. S2C, D). Previously, Choi et al. [50] demonstrated that the *Rpe65*^*CreERT2*^ homozygous mice exhibited reduced RPE65 isomerase levels compared to heterozygous and WT mice. However, we did not observe a decrease in RPE65 expression in our mice (Additional file 2: Fig. S2E). Nevertheless, since the ablation observed in the homozygous mice was comparable to that in the heterozygous mice (Additional file 2: Fig. S2F), we used the *Rpe65*^*CreERT2*^ heterozygous mice in our experiments.

The successful removal of miR-181a/b-1 at the genomic level, as evidenced by the predominant excised band in the agarose gel, was confirmed for each individual mouse. Subsequently, we evaluated the expression levels of miR-181a/b. As hypothesized, the treated group exhibited a decrease in both miR-181a and miR-181b expression at PW6 compared to the control group (Fig. 2B). Surprisingly, at PW10, we observed an upregulation of these miRs (Fig. 2C), contradicting our initial hypothesis. Since we previously showed dysregulated miR-181a/b expression in the disease background, we postulated if this downregulation at PW6 and upregulation at PW10 were also specific to the disease or present in the WT background. To investigate this, we generated the *Pde6β*^*WT*^ mice that are congenic with the *Pde6β*^*H620Q*^ mice. In the same litters, we obtained Pde6β^WT^miR-181a/b-1^+/+^ (WT control) and Pde6β^WT^miR-181a/b-1^−/−^ (WT treated) mice. The genomic excision of miR-181a/b-1, specifically in RPE cells, was also confirmed in these mice (Fig. 2D, and Additional file 2: Fig. S2C). Surprisingly, we found no changes in miR-181a/b expression in the RPE cells of WT treated or WT control mice at PW6 or PW10 (Fig. 2E, F). These results suggest that cluster 2 of miR-181a/b may play a compensatory role. Of particular significance, compensation effects in the *Pde6β*^*WT*^ mice are tightly regulated and only lead to reaching homeostatic levels without causing upregulation, as seen in the RPE cells of *Pde6β*^*H620Q*^ mice at PW10. This observation implies that the cellular compensatory system is dysregulated in the disease background.

**Fig. 3:**
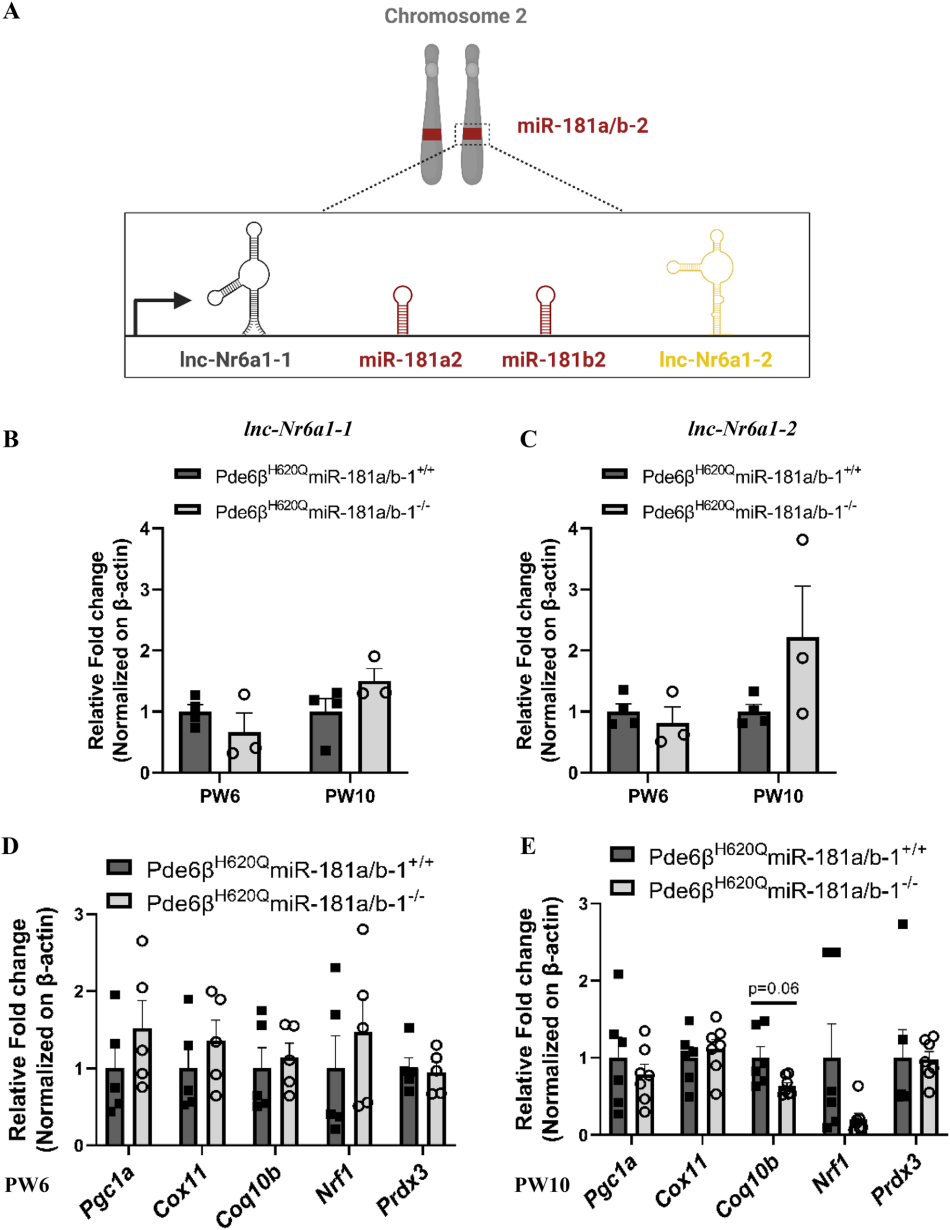
miR-181a/b-2 compensates the lost of miR-181a/b-1 in RPE cells of mutant mice. **A** Illustration showing that *lnc-Nr6a1* and miR-181a/b-2 share the same promoter. **B**–**C** qPCR analysis of (**B**) *lnc-Nr6a1-1* and **C** *lnc-Nr6a1-2* expression in the RPE cells of Pde6β^H620Q^miR-181a/b-1^+/+^ and Pde6β^H620Q^miR-181a/b-1^−/−^ mice at PW6 and PW10. n ≥ 3 mice/genotype. Data are presented as mean ± SEM. Student’s t-test, unpaired. **D**–**E** qPCR analysis of miR-181a/b targets expression in the RPE cells of Pde6β^H620Q^miR-181a/b-1^+/+^ versus Pde6β^H620Q^miR-181a/b-1^−/−^ mice at (**D**) PW6, and **E** PW10. n ≥ 4 mice/genotype. P-values are reported in black. Data are presented as mean ± SEM. Student’s t-test, unpaired with or without Welch ‘s correction

It has been previously observed that identical miRs encoded in different genomic regions can compensate for each other, meaning that the repression of one miR may lead to increased expression of its paralog [51]. Notably, miR‐181a/b-1 is encoded in the intron of a noncoding RNA host gene (*MIR181A1HG*), while miR‐181a/b-2 is encoded in the intron of the *Nr6a1* gene, which is also a host gene for a long non-coding RNA (lncRNA) known as *lnc-Nr6a1* [52, 53]. The processing of *lnc-Nr6a1* generates two polyadenylated isoforms, *lnc-Nr6a1-1* and *lnc-Nr6a1-2*, along with a non-polyadenylated lncRNA that further generates miR-181a-2 and miR-181b-2. Hence, *lnc-Nr6a1-1* and *lnc-Nr6a1-2* share the same promoter with miR-181a/b-2 (Fig. 3A) [53]. To support the hypothesis that miR-181a/b-2 compensates for the ablation of miR-181a/b-1 in the RPE cells of *Pde6β*^*H620Q*^ mice, we quantified the expression of *lnc-Nr6a1-1* and *lnc-Nr6a1-2* in the RPE cells of control and treated mice. We found that at PW6, the expression of *lnc-Nr6a1-1* and *lnc-Nr6a1-2* was similar between control and treated mice. However, at PW10, there is a trend showing a moderate increase in the expression of *lnc-Nr6a1-1* and *lnc-Nr6a1-2* (Fig. 3B, C). These findings suggest that compensatory mechanisms were probably not activated at PW6 but were already in effect by PW10. Next, we assessed how the ablation of miR-181a/b-1 affects the expression of some of their mRNA targets in the RPE cells of the *Pde6β*^*H620Q*^ mutant mice. We focused on targets involved in mitochondrial function, including *Nrf1*, *Cox11*, *Coq10b*, *Prdx3*, and *Pgc1α*, which were previously upregulated in the retina of miR-181a/b^−/−^ mice [36]. Although we did not find statistically significant changes, we observed a trend of increased transcript levels of miR-181a/b targets at PW6 (Fig. 3D), and the opposite trend at PW10 (Fig. 3E), as measured by quantitative (q)RT–PCR. These trends are consistent with our previous finding showing miR-181a/b downregulation at PW6 and upregulation at PW10. Taken together, these results showed that the ablation of miR-181a/b-1 in RPE cells of *Pde6β*^*H620Q*^ mice induces dynamic changes in miR-181a/b expression. We observe decreased levels at PW6 and increased levels at PW10, possibly linked to a compensatory mechanism exhibited by miR-181a/b-2. This dynamic miR-181a/b expression impacts the expression of miR-181a/b mRNA targets, showing an increasing trend at PW6 and a decreasing trend at PW10. Importantly, the dynamic nature of miR-181a/b expression, transitioning from downregulation at PW6 to upregulation at PW10, may contribute to subtle differences between control and treated mice. These dynamics complicate the detection of distinctions, resulting in trend observations rather than statistically significant differences.

### miR-181a/b downregulation in RPE cells transiently retards the retinal degeneration in ***Pde6β***^***H620Q***^ mice

Building upon our earlier discoveries indicating dysregulation of miR-181a/b in the RPE and NR of the *Pde6β*^*H620Q*^ mutant mice, as well as the observed dynamic pattern of miR-181a/b expression following tamoxifen injection with downregulation at PW6 and upregulation at PW10, we sought to investigate how this dynamic expression affects NR cells through disease progression. Specifically, we aimed to understand if the downregulation of miR-181a/b in RPE cells observed at PW6 would yield beneficial effects and conversely, whether the upregulation at PW10 would have adverse consequences on the disease course.

**Fig. 4:**
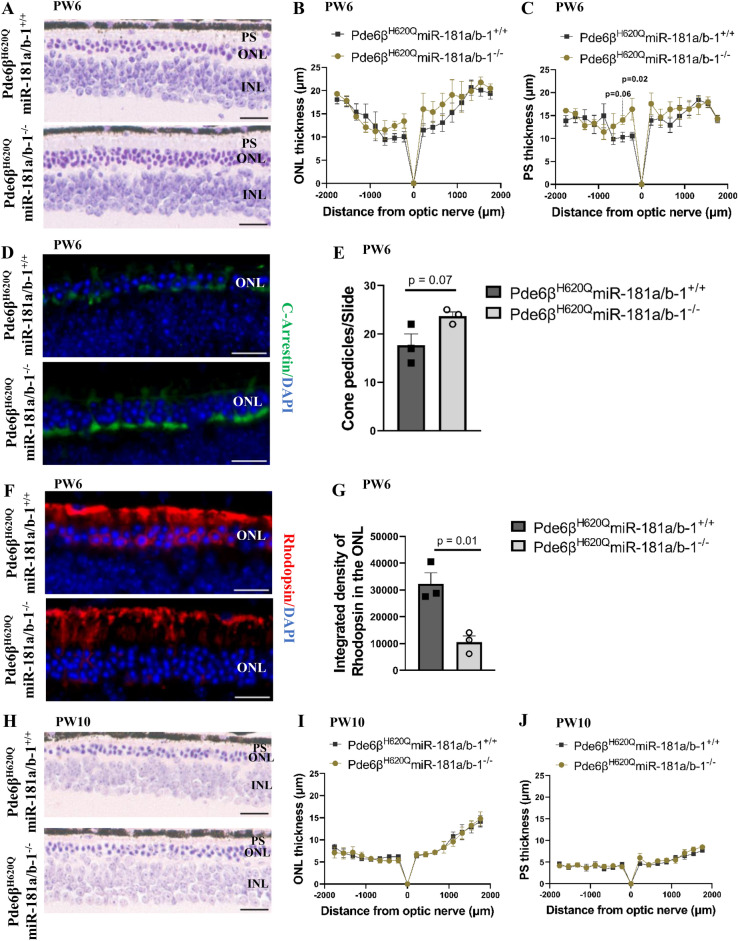
Transient miR-181a/b downregulation in the RPE cells of *Pde6β*^*H620Q*^* mice* at PW6 ameliorates disease phenotype. **A**–**C** Morphological analysis of Pde6β^H620Q^miR-181a/b-1^+/+^ and Pde6β^H620Q^miR-181a/b-1^−/−^ retinas at PW6. **A** Representative histological sections. Scale bar 20 µm. The thickness profiles of (**B**) PRs ONL and **C** PS across the whole retina. N ≥ 3 mice/genotype. Each dot represents the average of the thicknesses at the specified point. P-values are reported in black. Data are presented as mean ± SEM. Student’s t-test, unpaired. **D**–**G** Immunofluorescence analysis of (**D**–**E**) C-Arrestin; and **F**–**G** Rhodopsin in Pde6β^H620Q^miR-181a/b-1^+/+^ and Pde6β^H620Q^miR-181a/b-1^−/−^ retinas at PW6. Scale bar 20 µm in **D**–**F**. Number of cone pedicles quantified on the C-Arrestin slides are represented in (**E**). Fluorescence densitometry quantification of rhodopsin internalized into PRs nuclei layer is reported in (**G**). N = 3 mice/genotype. P-values are reported in black. Data are presented as mean SEM. Student’s t-test, unpaired. **H**–**J** Morphological analysis of Pde6β^H620Q^miR-181a/b-1^+/+^ and Pde6β^H620Q^miR-181a/b-1^−/−^ retinas at PW10. **H** Representative histological sections. Scale bar 20 µm. The thickness profiles of (**I**) PRs ONL and **J** PS across the whole retina. N ≥ 3 mice/genotype. Each dot represents the average of the thicknesses at the specified point. Data are presented as mean ± SEM. Student’s t-test, unpaired

To achieve this goal, we analyzed histological sections and performed ERGs on mice at PW6 and PW10. Previous studies have shown that these mice experience progressive PR degeneration with a gradual reduction in nuclei numbers in the outer nuclear layer (ONL) from P19 to P49, leaving only 1–2 rows of PR nuclei [39]. ERG responses also progressively decrease from P20 in both scotopic and photopic responses [39]. This condition, known as rod-cone dystrophy, leads to the initial degeneration of rod PR followed by cone PR degeneration, resulting in a persisting photopic response longer than the scotopic response [39]. Although we did not observe any changes in scotopic or photopic function measured by ERG at PW6 or PW10 (Additional file 3: Fig. S3A-G), we did notice morphological improvements in the PR segment (PS) and ONL thickness at PW6. Specifically, the treated group exhibited a tendency of increasing ONL thickness and statistically significant elongation of PS at the central retina near the optic nerve compared to the control group (Fig. 4A–C). Interestingly, at the central retina, the same area where we observed improved PS and ONL thickness at PW6, we found better preservation of cone PRs. The treated group showed an increased number of cone PR pedicles compared to controls (Fig. 4D, E and Additional file 4: Fig. S4A-D). Additionally, Rhodopsin immunohistochemistry revealed less cytoplasm internalization of rhodopsin and better maintenance of its prototypical localization in the rod PR outer segments in the treated group. Furthermore, PR outer segments appeared longer and better preserved in the treated group compared to the control at PW6 (Fig. 4F, G and Additional file 4: Fig. S4E-H). As hypothesized, the improvements in the morphological phenotype were not sustained up to PW10 (Fig. 4H–J). Importantly, the retinal morphology and function did not worsen with the miR-181a/b upregulation observed at PW10. Taken together, our findings suggest that the specific downregulation of miR-181a/b in RPE cells has beneficial effects and slows down retinal degeneration in *Pde6β*^*H620Q*^ mice at PW6. However, the miR-181a/b upregulation at PW10 did not lead to a worsening of the disease phenotype.

### miR-181a/b sustains aerobic glycolysis in RPE cells

**Fig. 5:**
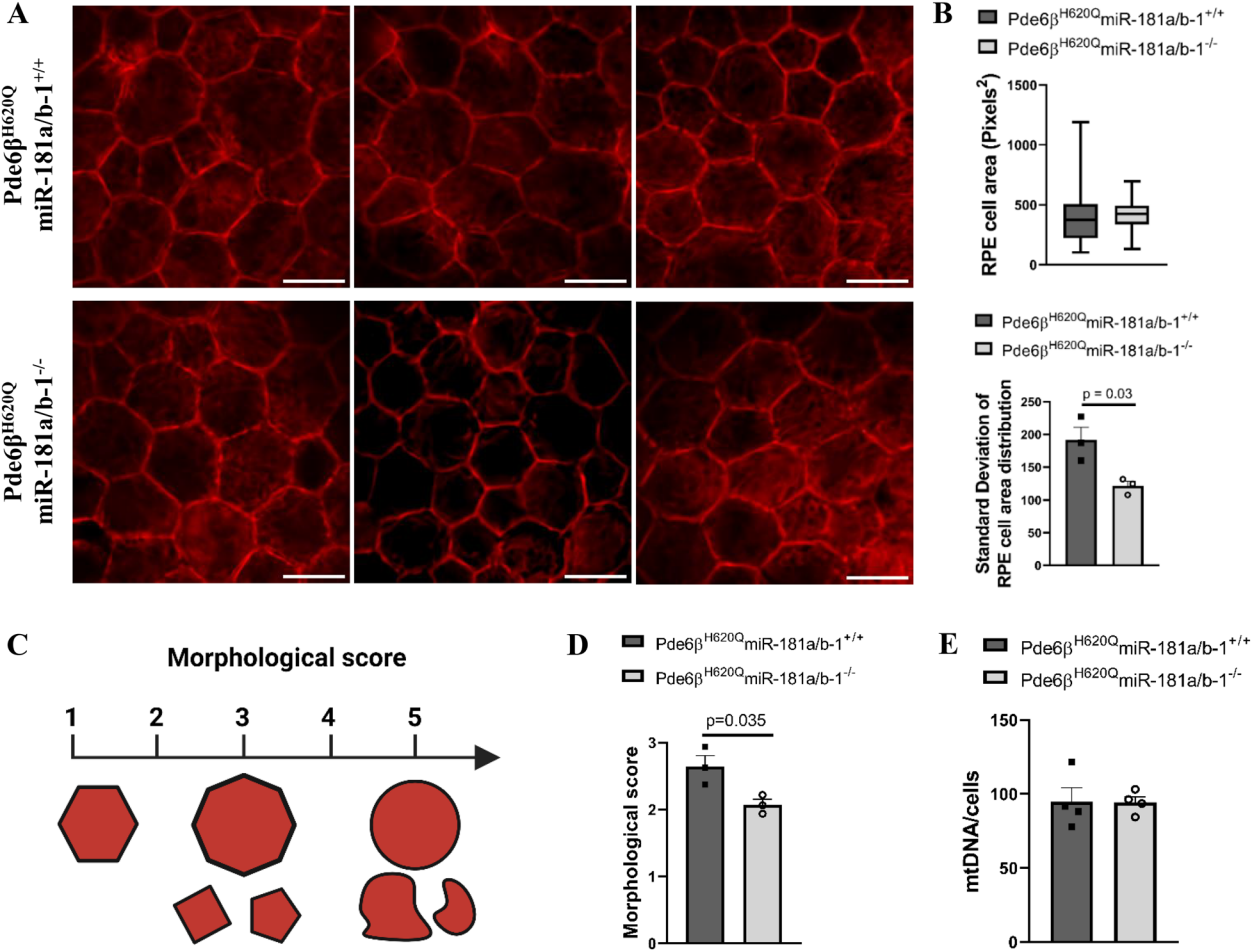
miR-181a/b downregulation ameliorated RPE morphology but did not increase mtDNA at PW6. **A**–**D** RPE morphometric analysis of the central area of RPE flat mounts using representative pictures of **A** Phalloidin staining from Pde6β^H620Q^miR-181a/b-1^+/+^ and Pde6β^H620Q^miR-181a/b-1^−/−^ mice at PW6. N = 3 mice/genotype. Scale bar 20 µm. **B** Quantification of RPE cell area (in pixels) in the upper panel shows a heterogeneous distribution of RPE cell area in the Pde6β^H620Q^miR-181a/b-1^+/+^ in comparison to Pde6β^H620Q^miR-181a/b-1^−/−^. In the lower panel, we further confirmed the larger variability of the RPE cell area distribution in Pde6β^H620Q^miR-181a/b-1^+/+^ versus Pde6β^H620Q^miR-181a/b-1^−/−^ mice. RPE cell area was quantified in six to eight areas of central RPE for each mice. N = 3 mice/genotype. In the upper panel data are presented as box plots showing the distribution of cell area for all RPE cells analyzed per group. In the lower panel, data are presented as mean ± SEM. Student’s t-test, unpaired. **C** Schematic showing how the health of RPE cells was scored. Scores 1 and 5 stand for the healthier and diseased morphology, respectively. **D** Qualitative scores attributed by two blinded observers to evaluate the RPE cells morphology of mice at PW6. N = 3 mice/genotype. Scores were attributed to four images of central RPE for each mice. Data are presented as mean of scores per mice ± SEM. Student’s t-test, unpaired. **E** mtDNA content measured by qPCR from Pde6β^H620Q^miR-181a/b-1^+/+^ and Pde6β^H620Q^miR-181a/b-1^−/−^ eye cups at PW6. N = 4 mice/genotype. Data are presented as mean ± SEM. Student’s t-test, unpaired

In this study, we modulated miR-181a/b expression exclusively in the RPE cells and achieved miR-181a/b downregulation only at PW6. We demonstrated that the downregulation of miR-181a/b at PW6 yields positive outcomes for NR cells. Subsequently, we explored its impact on the well-being of RPE cells. Previous work by Carrela et al. demonstrated that simultaneous downregulation of miR-181a/b in multiple retinal cells, including rod PRs and RPE cells, led to the most effective rescue of RP phenotype, which was attributed to improved mitochondrial function [18]. The downregulation of miR-181a/b observed at PW6 has the potential to enhance RPE mitochondrial function, which is crucial for maintaining RPE health, as these cells heavily rely on mitochondria to meet their high energetic demands [8, 27–29]. RPE morphology serves as a critical parameter in assessing cellular health. Healthy RPE cells display a well-organized monolayer of hexagonal shape with uniform size. In contrast, RPE monolayers exhibit spatial irregularities in retinal diseases, losing their characteristic hexagonal shape and exhibiting irregular and heterogeneous sizes and shapes [54]. PR degeneration drives RPE morphology changes in different RP models [2, 47]. We examined RPE morphology at PW6 to assess whether miR-181a/b downregulation correlates with improvements in RPE cell morphology. As hypothesized, the central RPE cells of the treated group displayed an improved morphology compared to the control group. Although we did not observe a significant difference between the average RPE cell area between control and treated groups, we did notice that the treated mice exhibited a more homogenous distribution of RPE cells with more uniform size and shape, whereas control mice showed several RPE cells with increased or decreased size, polymegathism and non-hexagonal shape (Fig. 5A, B). Further, the scoring of morphological aspects of RPE cells confirmed statistical differences between the control and treated groups (Fig. 5C, D).

*Nrf1* and *Pgc1a* are the targets that showed the most considerable increase in expression with the downregulation of miR-181a/b in RPE cells at PW6. Both targets are key regulators of mitochondrial biogenesis [55–57]. Essentially, members of the *Pgc1* family stimulate the expression of NRF1 and activate its transcription factor function, resulting in mitochondrial DNA (mtDNA) replication [55]. We tested if the mtDNA content increases upon downregulation of miR-181a/b in RPE cells at PW6. Surprisingly, no changes in mtDNA were observed at PW6 (Fig. 5E). However, it is important to note that miR-181a/b downregulation has been previously associated with improvements in mitochondrial function without necessarily leading to increased mtDNA [18].

**Fig. 6:**
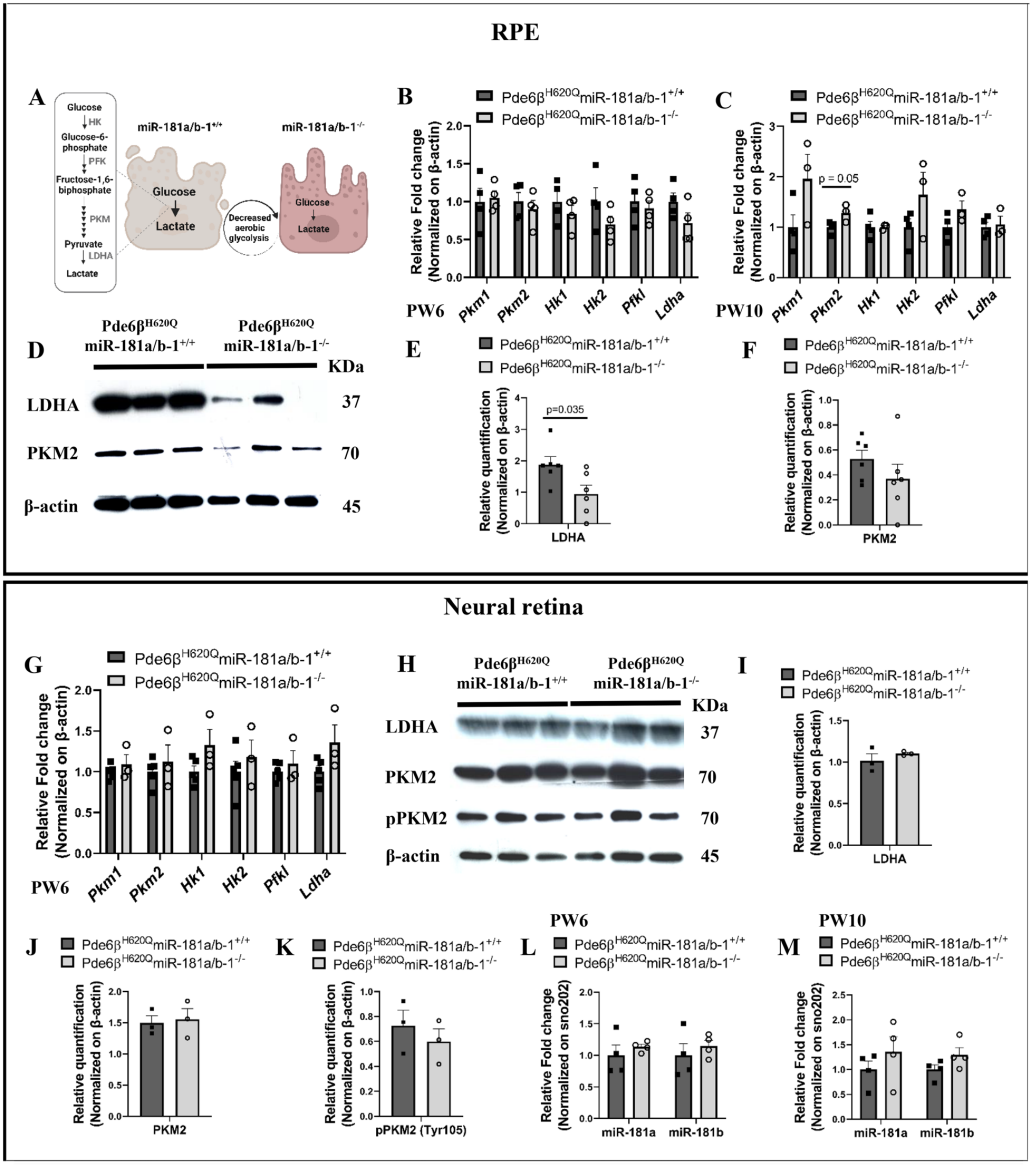
LDHA expression decreases with downregulation of miR-181a/b in the RPE of *Pde6β*^*H620Q*^ mice at PW6.** A** Illustration of the proposed hypothesis: miR-181a/b downregulation makes RPE cells healthier and decreases the aerobic glycolysis in those cells. **B**–**C** qPCR analysis of the expression of key regulators of aerobic glycolysis in the RPE cells of Pde6β^H620Q^miR-181a/b-1^+/+^ and Pde6β^H620Q^miR-181a/b-1^−/−^ mice at (**B**) PW6, and **C** PW10. N ≥ 3 mice/genotype. Data are presented as mean ± SEM. Student’s t-test, unpaired. **D**–**F** Western Blotting analysis in (**D**) reveals decreased levels of LDHA protein in the RPE cells of Pde6β^H620Q^miR-181a/b-1^−/−^ versus Pde6β^H620Q^miR-181a/b-1^+/+^. Data are normalized to β-actin. N = 3 mice/genotype. Please note that all compared bands are from the same blots. LDHA bands are quantified in (**E**) and PKM2 bands in (**F**). N = 6 mice/genotype. Data are presented as mean ± SEM. Student’s t-test, unpaired. **G**–**K** Analysis of the expression of key regulators of aerobic glycolysis at (**G**) mRNA and **H**–**K** protein levels in the NR of Pde6β^H620Q^miR-181a/b-1^+/+^ and Pde6β^H620Q^miR-181a/b-1^−/−^ mice at PW6. Data are presented as mean ± SEM for N ≥ 3 mice/genotype and Student’s t-test, unpaired in (**G**). Please note that all compared bands are from the same blots in (**H**), LDHA bands are quantified in (**I**), PKM2 bands are quantified in (**J**), and pPKM2 (Tyr105) bands are quantified in (**K**). N = 3 mice/genotype. Data are presented as mean ± SEM. Student’s t-test, unpaired. (**L**–**M**) qPCR analysis reveals that miR-181a/b expression does not change in the NR of Pde6β^H620Q^miR-181a/b-1^−/−^ in comparison to Pde6β^H620Q^miR-181a/b-1^+/+^ at (**L**) PW6, and **M** PW10. N = 4 mice/genotype. Data are presented as mean ± SEM. Student’s t-test, unpaired

Furthermore, *Pgc1a*, in addition to regulating mitochondrial biogenesis, inhibits aerobic glycolysis and upregulates OXPHOS of RPE cells both in vitro and in vivo [58–60]. The metabolic state of RPE cells indirectly reflects the health of their mitochondria, where healthy RPE cells predominantly rely on OXPHOS and reductive carboxylation, processes highly dependent on mitochondrial function. In contrast, diseased RPE cells exhibit increased aerobic glycolysis [8, 27–29]. We next investigated whether miR-181a/b downregulation decreased aerobic glycolysis in RPE cells of *Pde6β*^*H620Q*^ mice at PW6 (Fig. 6A). While no statistically significant changes were evident, there was a noticeable trend indicating reduced expression at the mRNA level for key regulators of aerobic glycolysis (Fig. 6B). The opposite was observed at PW10, including a significant pyruvate kinase M2 (*Pkm2*) increase in the treated group (Fig. 6C). The lack of correlation between mRNA and protein expressions is a well-established observation in scientific studies [61–63]. Various factors can disrupt the linear relationship between mRNA and protein levels, such as intricate events occurring between transcription and translation, as well as differences in the degradation and synthesis ratios of proteins and mRNAs [61]. Consequently, minor changes at the mRNA level may result in significant alterations at the protein level. In light of this, we assessed the protein levels of LDHA and PKM2, which are downstream regulators of the aerobic glycolysis pathway. We did not observe changes in *PKM2* expression in the RPE cells between control and treated mice. However, in RPE cells of treated mice, where there is a downregulation of miR-181a/b, we observed significantly reduced levels of LDHA (Fig. 6D–F).

Given the evidence suggesting that the downregulation of miR-181a/b in RPE cells resulted in decreased LDHA levels, indicating a likely reduction in RPE glycolytic activity, we aimed to explore whether this decrease in aerobic glycolysis in RPE cells had an impact on the glycolytic activity in the neighboring neural retina (NR) at PW6. We observed a trend of increased *Ldha* expression at the mRNA level (Fig. 6G), but no significant differences were found in LDHA, PKM2, and pPKM2 expression at the protein level (Fig. 6H–K). A previous study by Carrella et al. showed that downregulating miR-181a/b, specifically in rod PRs, improved the retinal disease phenotype in P347S mice [18]. Since we did not observe changes in the glycolytic state of NR at PW6, we hypothesize that miR-181a/b may be transferred from RPE to NR via extracellular vesicles, resulting in direct changes in miR-181a/b expression in NR cells. However, no changes in miR-181a/b expression were detected in NR cells upon ablation of miR-181a/b-1 in RPE cells at PW6 or PW10 (Fig. 6L, M). Taken together, these results suggest that miR-181a/b downregulation in RPE cells makes those cells healthier and less glycolytic.

## Discussion

This study aimed to investigate the expression pattern of miR-181a/b during the progression of RP in both the NR and RPE cells of a preclinical *Pde6β*^*H620Q*^ mouse model. Surprisingly, we observed opposite expression profiles of miR-181a/b in these tissues. In the NR, miR-181a/b was found to be decreased at the onset of degeneration (PW3), but it showed a significant increase as the disease progressed. On the other hand, in RPE cells, we observed a consistent decrease in miR-181a/b expression. Specifically, miR-181b remained stably decreased since the early stages of the disease (PW3), while miR-181a decreased throughout disease progression. The disease-associated molecular and cellular remodeling process can manifest in either degradative or constructive forms [14, 17]. Prior research has indicated that degradative remodeling can exacerbate the disease phenotype [64–66], whereas constructive remodeling may act as a compensatory mechanism to slow down cell death [67, 68]. The dysregulated expression of miR-181a/b in NR and RPE cells suggests a complex interplay of remodeling processes during the progression of PR degeneration in *Pde6β*^*H620Q*^ mice. Earlier studies showed that decreasing miR-181a/b levels in rod PRs improved mitochondrial function and rescued the RP phenotype in P*de6β*^*rd10*^ mice [18], which bear a missense mutation in the catalytic domain of the *Pde6β* gene, similar to *Pde6β*^*H620Q*^ mice. This finding suggests that the decreased levels of miR-181a/b in the NR of *Pde6β*^*H620Q*^ mice at PW3 might serve as a compensatory mechanism to protect against PR death. However, as the disease progresses, the degeneration of PRs impairs their compensatory capacity, resulting in the upregulation of miR-181a/b, which supports disease progression. Notably, the RPE cells have also been found to undergo remodeling in *Pde6β*-associated RP, believed to be a constructive remodeling process that aids in slowing down PR degeneration and restoring cellular homeostasis [47]. In our study, we observed that the downregulation of miR-181a/b in RPE at PW6 enhanced the survival of the degenerating retina in *Pde6β*^*H620Q*^ mice. Furthermore, Carrela et al. [18] demonstrated that simultaneous reduction of miR-181a/b levels in various retinal cells, including rod PRs and RPE cells, resulted in the most effective rescue of the RP phenotype. Collectively, these findings support the hypothesis that the molecular remodeling observed in RPE cells in our study is likely a naive self-defense compensatory mechanism aimed at combatting and preventing cell death in the context of RP progression.

We applied a Cre-LoxP system to investigate the specific effects of miR-181a/b-1 ablation in the progression of RP. Our results demonstrated that tamoxifen efficiently facilitated the excision of miR-181a/b-1 in the majority of RPE cells in all experimental mice. However, we only observed miR-181a/b downregulation in the RPE cells of *Pde6β*^*H620Q*^ mice at PW6. Surprisingly, at PW10, we observed an upregulation of miR-181a/b. Importantly, miR-181a/b-1 excision in the RPE cells of *Pde6β*^*WT*^ mice did not lead to any changes in miR-181a/b levels at either PW6 or PW10. These findings suggest that miR-181a/b-2 may compensate for the ablation of miR-181a/b-1, which is further indicated by the trend of increased expression of *Nr6a1-1* and *lnc-Nr6a1-2* transcripts in the RPE cells of *Pde6β*^*H620Q*^ mice at PW10. Compensation of miRs that have similar seed sequences and target genes has been previously discussed in the retina. For instance, Jin et al*.* demonstrated that depleting miR-182 in the mouse retina did not lead to significant transcriptional or phenotypic changes, possibly due to in vivo compensation from miR-183 and miR-96, which share similar seed sequences and mRNA targets as miR-182 [69]. Interestingly, miR-181a/b-1 depletion in the mouse retina of miR-181a/b-1^−/−^ mice and in other tissues, such as Kras-dependent lung tumors, did not show evidence of compensation [36, 70]. However, our novel findings indicate that miR-181a/b-2 compensates for the loss of miR-181a/b-1 specifically in RPE cells, highlighting that this compensatory process might be cell-specific. The crucial role of miR-181a/b in regulating vital pathways in vivo was previously explored [49], and our results in WT mice emphasize the significance of miR-181a/b in normal RPE function. The lack of changes in miR-181a/b levels upon cluster 1 ablation in the RPE cells of *Pde6β*^*WT*^ mice suggests the existence of a highly specialized and optimized feedback system in these cells, ensuring that one cluster compensates for the other to maintain cellular homeostasis. Nevertheless, this feedback system becomes dysregulated in the disease context, disrupting this precise compensatory mechanism. Consequently, we observed the unexpected upregulation of miR-181a/b in the RPE cells of *Pde6β*^*H620Q*^ mice at PW10. These results shed light on the intricate regulatory mechanisms involving miR-181a/b in retinal degeneration and may provide insights into the mechanisms of therapeutic strategies previously explored in the field [18].

We observed that miR-181a/b downregulation exerts a beneficial effect in a mouse model of RP at PW6. We found that this downregulation resulted in anatomical improvements, characterized by a higher density of cones PR, longer PR segments, and healthier rhodopsin distribution in rod outer segments. Similar findings were also reported by Carrella et al. [18] in the P347S mouse model. Importantly, it is essential to note that in their study, the downregulation of miR-181a/b was performed in the entire retina, affecting multiple retinal cell types. Furthermore, in their model, miR-181a/b downregulation initiated during embryonic development, suggesting that the rescue effect could be influenced by the combined impact of miR-181a/b downregulation in various retinal cells and the effects during early developmental stages, possibly before P8 (the time point at which we induced miR-181a/b downregulation in our study). Carrella et al. [18] also investigated the effects of miR-181a/b downregulation through subretinal delivery of AAV2/8.CMV.GFP-Sponge-miR-181a/b in the *Pde6β*^*rd10*^ mouse model. They observed functional rescue only when the injections were performed at P4 using the ubiquitous CMV promoter, further supporting the hypothesis that the beneficial effects of miR-181a/b downregulation might be associated with early and combined downregulation of miR-181a/b in multiple retinal cell types.

In particular, our findings suggest that the downregulation of miR-181a/b, specifically in RPE cells, plays a critical role in delaying PR degeneration in RP. Carrella et al. [18] reported improved PR function (up to P70) by delivering AAV2/8.CMV.GFP-Sponge-miR-181a/b in vivo. The “sponge” used in their study is a miR-181a/b inhibitor sequence that targets both miR-181a/b-1 and miR-181a/b-2, thus circumventing miR compensation effects and allowing for long-lasting rescue effects. The crucial point to highlight is that the AAV2/8.CMV.GFP-Sponge-miR-181a/b construct was highly expressed in RPE cells. Our study provides compelling evidence that the downregulation of miR-181a/b, specifically in RPE cells at PW6, retards PR degeneration in a mouse model of RP. This data supports the notion that achieving the optimal therapeutic effect, as demonstrated by Carrella et al. in their study [18], necessitates miR-181a/b downregulation in RPE cells. However, it is crucial to consider the delivery approach for miR regulation, as previous studies have shown that the administration of AAV8-CMV-GFP to the retina of mice can lead to toxicity in PRs and RPE, as well as glial cell activation. Toxicity was observed using broadly active promoters like the CMV or RPE-specific promoter [71]. Therefore, while downregulating miR-181a/b in various retinal cell types may be beneficial, the delivery approach warrants further investigation. As a therapeutic paradigm/methodology, the “sponge” approach appears to be the ideal strategy for regulating miR expression, especially for the miRs organized in different clusters in mammalian cells that could display compensatory effects. However, this approach may skew the mechanistic understanding of the compensatory mechanisms involved in the cell-specific modulation of miR expression, which might be essential for understanding the pathogenesis of disease progression.

**Fig. 7:**
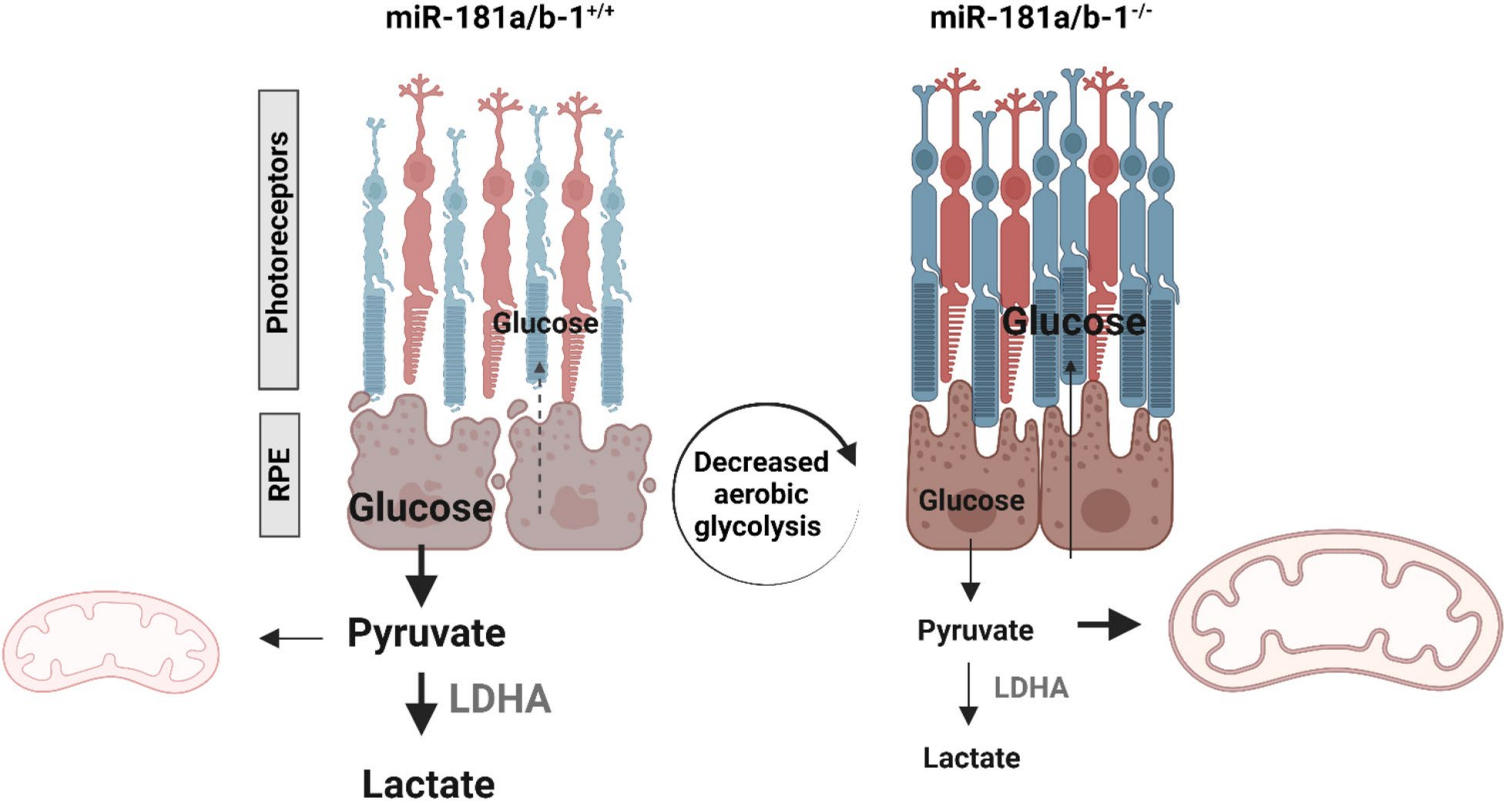
miR-181a/b sustains aerobic glycolysis in RPE cells. Reduced expression of miR-181a/b at PW6 enhances the vitality of RPE cells. Consequently, these cells shift away from utilizing glucose via aerobic glycolysis for energy generation, favoring OXPHOS instead. This shift implies a potential increase in glucose availability for PR

Early studies investigating the therapeutic potential of miR-181a/b downregulation for inherited retinal diseases (IRDs) revealed improvements in disease phenotype associated with enhanced mitochondrial morphology and function. These improvements included increased mitochondria biogenesis and turnover, and reduced mitochondrial fragmentation [18, 36]. However, the role of miR-181a/b in regulating the metabolism of retinal cells has not been previously explored. Our study shed light on this aspect and demonstrated that miR-181a/b downregulation in RPE cells improved their morphology and reduced their glycolytic activity. This decrease in aerobic glycolysis in RPE cells is particularly significant for treating IRDs as it suggests a higher availability of glucose for PRs, which is known to be beneficial [33–35]. In the healthy state, LDHA is prominently expressed in glycolytic cancer cells and PRs, where it converts pyruvate to lactate, the final step of aerobic glycolysis. Conversely, LDHB is highly expressed in the inner retina and RPE cells, performing the opposite conversion of lactate to pyruvate [72, 73]. Our results at PW6 revealed high levels of LDHA in the control group, while the treated group exhibited decreased levels. These findings suggest that the downregulation of miR-181a/b in RPE cells of *Pde6β*^*H620Q*^ mice reduced the glycolytic activity within RPE cells, suggesting a decrease in glucose consumption by the RPE. LDHA produced NAD^+^, the rate-limiting substrate for glycolysis. Consequently, decreased RPE glycolysis would permit more choroidal glucose to support PRs, potentially contributing to their improved function and survival (Fig. 7). Surprisingly, the expression of *LDHA* and *PKM2* did not show any upregulation in the retina. However, this does not necessarily imply that aerobic glycolysis is not affected in the retina. It is plausible that although the expression of key enzymes involved in aerobic glycolysis remains unchanged, the downregulation of miR-181a/b in RPE cells could result in increased glucose availability for PRs. This surplus of glucose could accelerate the rate of aerobic glycolysis without necessitating an increase in the expression of glycolytic enzymes. In this context, the glycolytic flux in PRs might be enhanced, leading to higher consumption and production of essential metabolites. Such alterations in the metabolic state of PRs could restore homeostasis, thereby mitigating the progression of the disease. While the precise mechanisms underlying this phenomenon require further investigation, our findings suggest that miR-181a/b downregulation in RPE cells may impact PR metabolism and overall retinal health in the context of retinal disorders.

This study was driven by three main objectives: (1) to examine the expression pattern of miR-181a/b during the progression of RP; (2) to investigate the role of miR-181a/b downregulation specifically in RPE cells in preserving PRs from degeneration in RP, as suggested by Carrella et al. in their study [18]; (3) to explore if the benefits of miR-181a/b downregulation in RPE cells, in terms of preserving PRs, are associated with improvements in RPE morphology and glycolytic state. Our findings revealed dysregulation of miR-181a/b in the NR and RPE cells of RP mice. Increased miR-181a/b levels in the NR during PR degeneration seem to contribute to the progression of the disease. Conversely, the downregulation of miR-181a/b in RPE cells appears to have a compensatory effect, protecting PRs from degeneration. These results shed light on the mechanistic basis for the therapeutic benefits observed in previous studies [18, 36]. Specifically, they offer an explanation for the observation that the most effective therapeutic outcomes were achieved using the “sponge” driven by a ubiquitous promoter rather than cell-specific promoters.

In the *Pde6β*^*H620Q*^ RP model, miR-181a/b downregulation in RPE cells improved the RP phenotype. Despite no observed changes in mtDNA content in the RPE cells, our results showed improved morphology and reduced aerobic glycolysis in these cells. This indicates that miR-181a/b downregulation may not increase the number of mitochondria but could enhance their function, as previously reported [18]. One indication of improved mitochondrial function is the reduction in aerobic glycolysis, suggesting that RPE cells are adopting healthier energy production mechanisms, such as mitochondrial oxidative phosphorylation and reductive carboxylation [27, 28]. However, the exact mechanisms underlying the suppression of aerobic glycolysis in RPE cells due to miR-181a/b downregulation require further investigation.

## Conclusion

Our research delves into the intricate mechanisms involved in the manipulation of miR-181a/b expression, shedding light on the metabolic pathways associated with its targeted reduction specifically within the RPE. Through our investigation, we uncover the understanding of the metabolic dynamics influenced by the downregulation of miR-181a/b. Furthermore, our findings underscore the significance of compensatory regulatory mechanisms among miR clusters, revealing a complex interplay within cellular processes. This insight not only enriches our comprehension of miR functionality but also holds significant implications for the design and development of miR-based therapeutic strategies.

### Supplementary Information

Additional file 1 (DOCX 21 KB)

Additional file 2 (PDF 701 KB)

Additional file 3 (DOCX 22 KB)

## Data Availability

The original data presented in this study are included in the article/additional files, and further inquiries can be directed to the corresponding authors.
