# Supplementary material for: Targeting miR-181a/b in retinitis pigmentosa: implications for disease progression and therapy
Source: Cell Biosci. 2024 May 21;14:64. doi: 10.1186/s13578-024-01243-3 (PMC11110387; doi:10.1186/s13578-024-01243-3)
Supplement: Supplementary file 1 — Additional file 1 (DOCX 21 KB) [file 13578_2024_1243_MOESM1_ESM.docx]

**Additional file 1: Fig. S1 Expression of miR-181a and miR-181b are dysregulated in the NR and RPE of *Pde6β^H620Q^* mice. (A-D)** qPCR analysis of **(A and C)** miR-181a and **(B and D)** miR-181b expression in the NR of **(A-B)** *Pde6β^WT^* and **(C-D)***Pde6β^H620Q^* mice at PW3, PW6, and PW10. N = 4 mice. **(E-H)** qPCR analysis of **(E and G)** miR-181a and **(F and H)** miR-181b expression in the RPE cells of **(E-F)***Pde6β^WT^* and **(G-H)***Pde6β^H620Q^* mice at PW3, PW6, and PW10. N ≥ 4 mice. P-values are reported in black. Data are presented as mean ± SEM. Student’s t-test, unpaired.

**Additional file 2: Fig. S2 *Rpe65^CreERT2^* mouse shows normal retina function and robust miR-181a/b excision in the RPE cells of mutant mice. (A)** Scotopic and Photopic ERG responses of Rpe65^+/+^(WT), Rpe65^+/CreERT2^(heterozygous), and Rpe65^CreERT2^(homozygous)  mice measured at 5 months-old. N = 3, 3 and 7 for WT mice. Data are presented as mean ± SEM. One-way ANOVA. **(B)** Representative histological sections of Rpe65^+/+^ (WT), Rpe65^+/CreERT2^ (heterozygous), and Rpe65^CreERT2^ (homozygous) retinas from 2 months-old mice. N = 3 mice/genotype. Scale bar 50µm. **(C)**Representative agarose gel electrophoresis result of genomic PCR to confirm absence of miR-181a/b-1 ablation in the NR of *Pde6β^H620Q^*and*Pde6β^WT^*mice.**(D)**Representative dideoxy sequencing chromatogram with the deleted 306bp band. **(E)** Western Blotting analysis of RPE65 expression in the RPE cells of RPE65^+/+^(WT), RPE65^+/CreERT2^ (heterozygous), and RPE65^CreERT2^(homozygous) mice. **(F)**Representative agarose gel electrophoresis result of genomic PCR showing comparable miR-181a/b-1 excision between RPE65^+/CreERT2^ (heterozygous) and RPE65^CreERT2^(homozygous) mice.

**Additional file 3: Fig. S3 Dynamic miR-181a/b expression in the RPE cells of *Pde6β^H620Q^* mice do not affect PRs function. (A-G)** ERG responses of Pde6β^H620Q^miR-181a/b-1^+/+^ and Pde6β^H620Q^miR-181a/b-1^-/-^ mice measured at **(A-D)** PW6 and **(E-G)** PW10. Scotopic b-wave in **(A)** was measured only for PW6. At PW10 there was no signal to record. Maximum response a-wave in **(B and E**); Maximum response b-wave in **(C and F)**; Photopic b-wave in **(D and G)**. N ≥ 12 mice/genotype. Data are presented as mean SEM. Student’s t-test, unpaired.

**Additional file 4: Fig. S4 miR-181a/b downregulation in the RPE cells of *Pde6β^H620Q^* mice improves PR morphology at PW6.** **(A-H)** Immunofluorescence analysis of **(A-D)** C-Arrestin; and **(E-H)** Rhodopsin in the two extra **(A, B, E, and F)** Pde6β^H620Q^miR-181a/b-1^+/+^ and **(C, D, G, and H)** Pde6β^H620Q^miR-181a/b-1^-/-^ mice at PW6. Scale bar 20µm.
