# Supplementary material for: Targeting miR-181a/b in retinitis pigmentosa: implications for disease progression and therapy
Source: Cell Biosci. 2024 May 21;14:64. doi: 10.1186/s13578-024-01243-3 (PMC11110387; doi:10.1186/s13578-024-01243-3)
Supplement: Supplementary file 2 — Additional file 2 (PDF 701 KB) [file 13578_2024_1243_MOESM2_ESM.pdf]

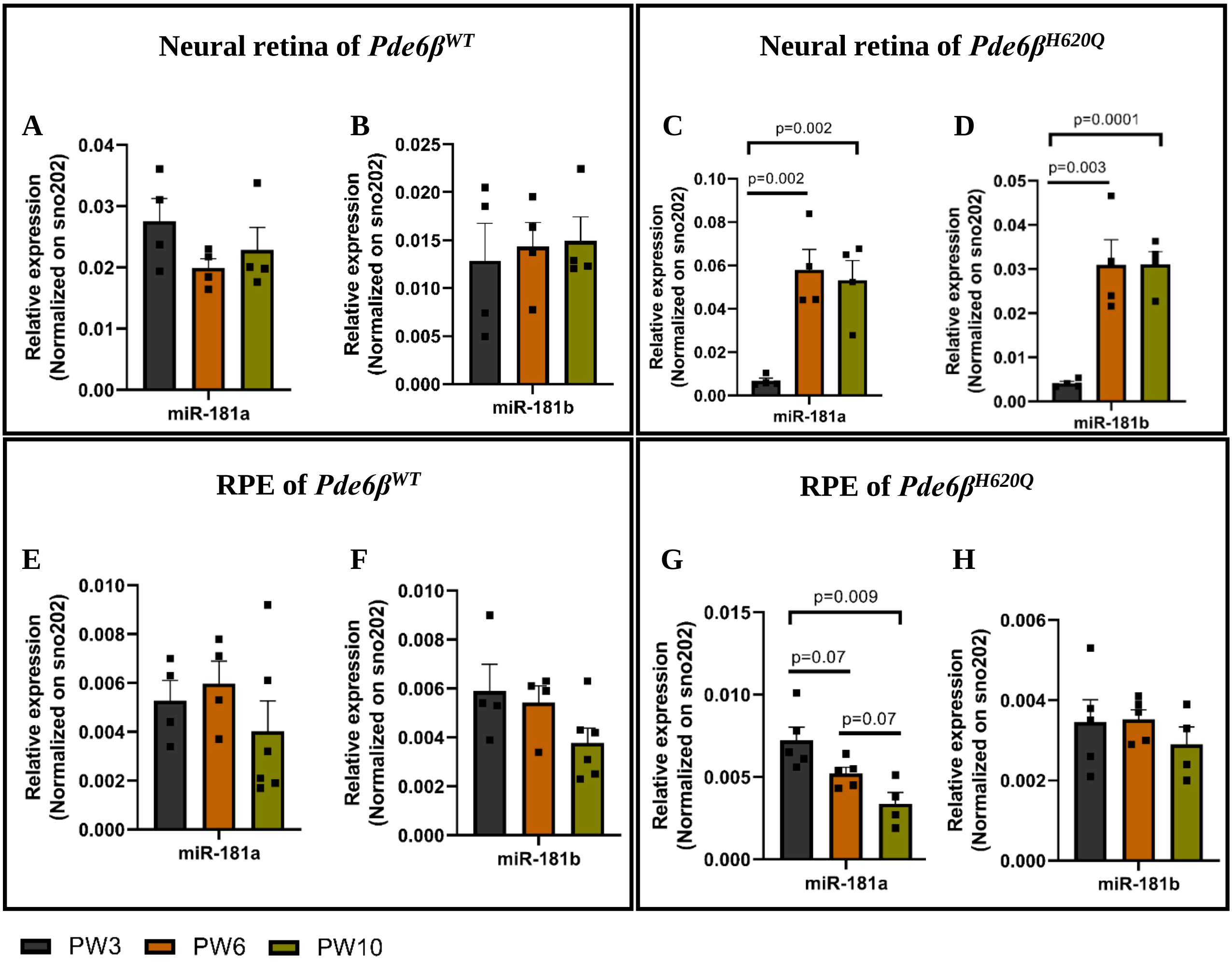

Additional file 1: Fig. S1

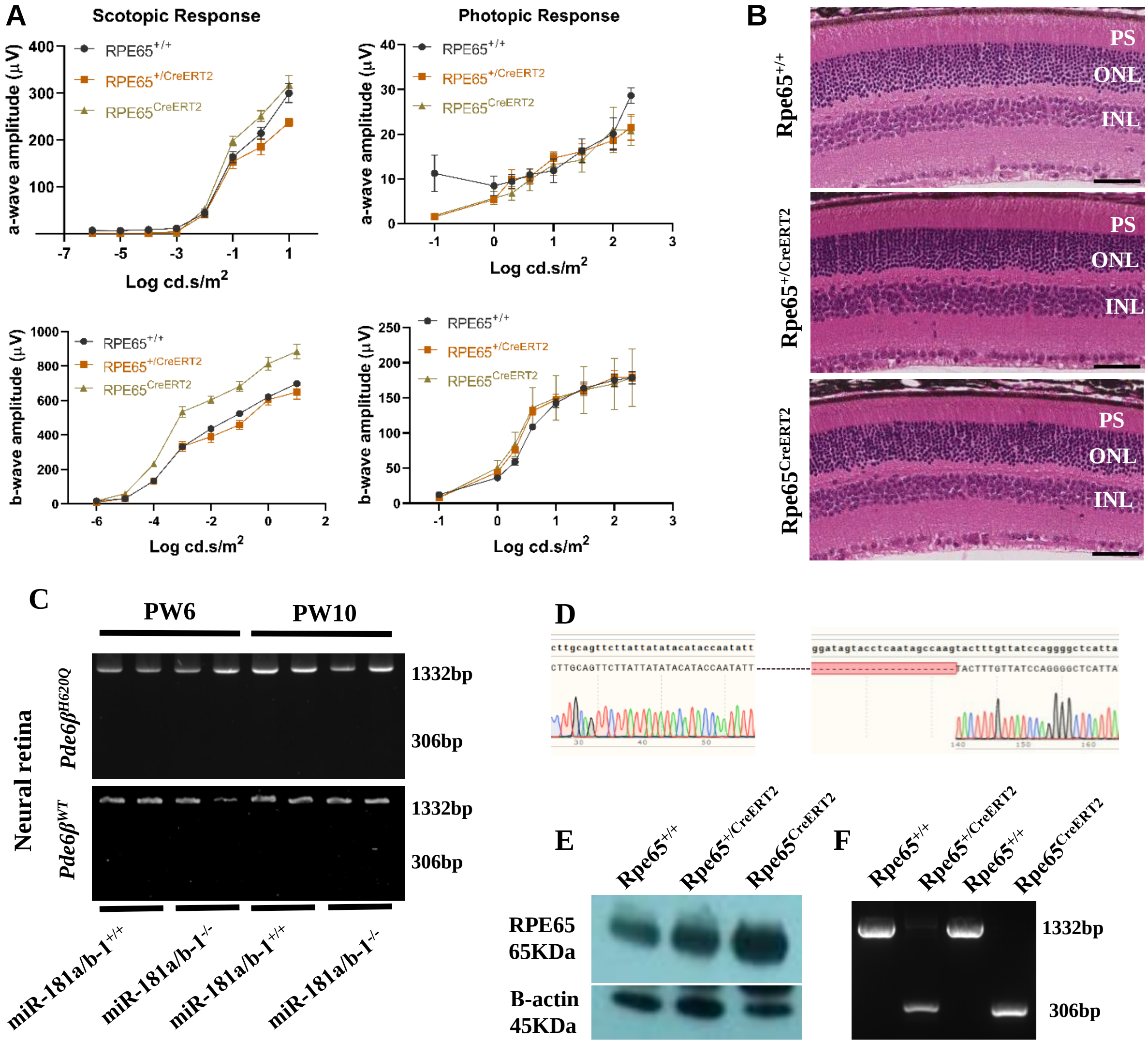

Additional file 2: Fig. S2

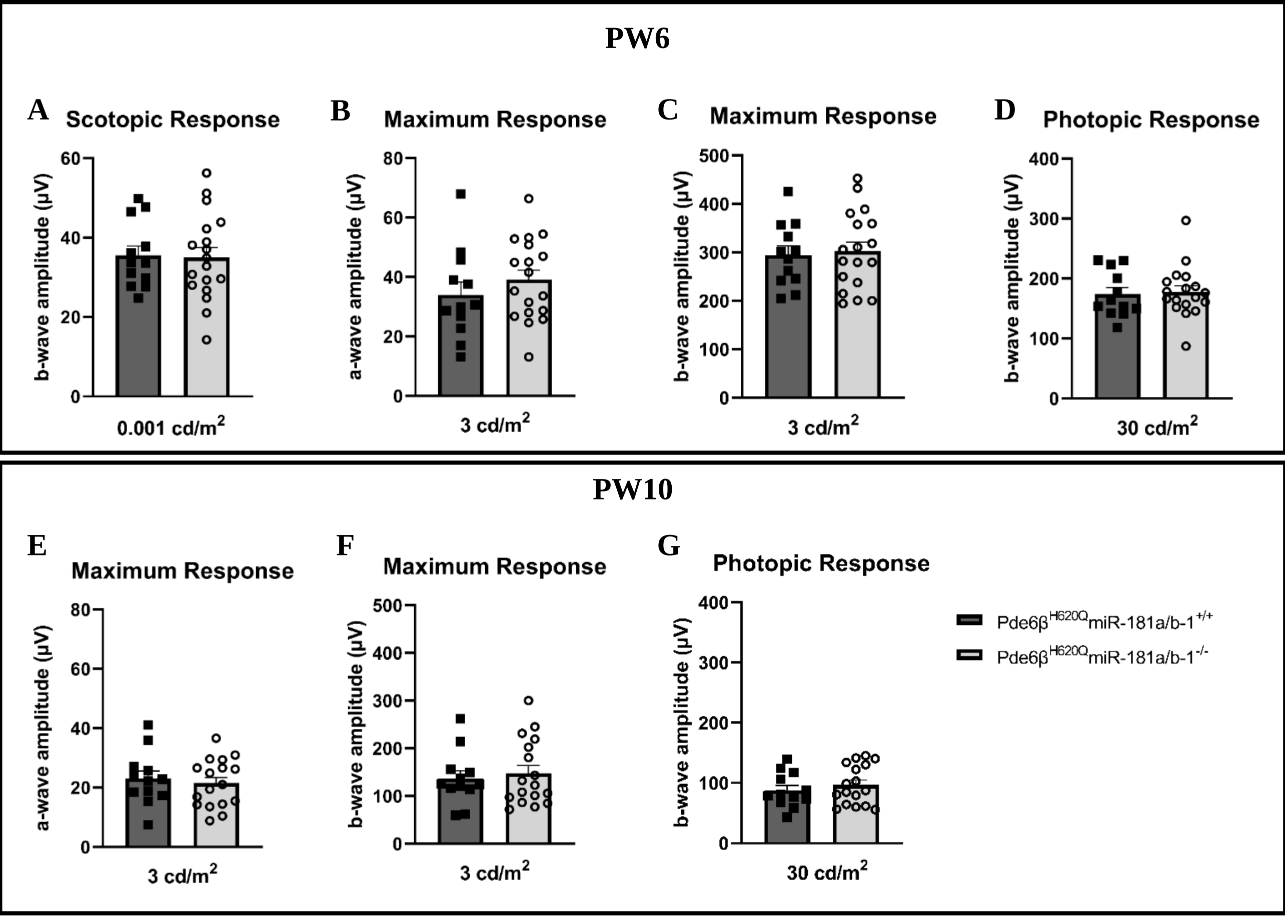

Additional file 3: Fig. S3

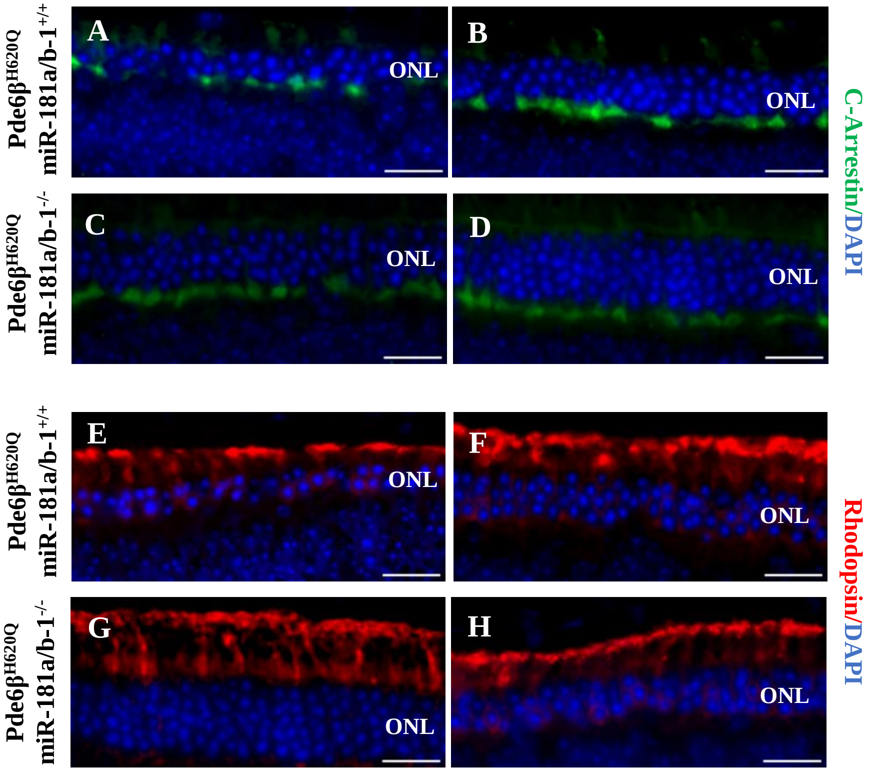

Additional file 4: Fig. S4
