# Supplementary material for: Targeting miR-181a/b in retinitis pigmentosa: implications for disease progression and therapy
Source: Cell Biosci. 2024 May 21;14:64. doi: 10.1186/s13578-024-01243-3 (PMC11110387; doi:10.1186/s13578-024-01243-3)
Supplement: Supplementary file 3 — Additional file 3 (DOCX 22 KB) [file 13578_2024_1243_MOESM3_ESM.docx]

**Table S1.** Primer sequences for qRT-PCR analysis.

| **Target** | **Forward** | **Reverse** |
| --- | --- | --- |
| *mmu-β-actin* | GAGGTCGACATTCACCATTC | AAGCTGTAGCCACGCTCGGTCA |
| *mmu-Pgc1a* | GGAATGCACCGTAAATCTGC | TTCTCAAGAGCAGCGAAAGC |
| *mmu-Nrf1* | AGCACGGAGTGACCCAAAC | TGTACGTGGCTACATGGACCT |
| *mmu-Cox11* | GAATCCTACTGACAAACCAG | GAGGTCGACATTCACCATTC |
| *mmu-Coq10b* | GATGATCATGGCAGCTCGGA | CTCGCACAGATCTCTTTAGG |
| *mmu-Prdx3* | GGAGTATTTCTGCCTCAACAG | CTCTCCATTGACAACAGCAG |
| *mmu-Hk1* | CGTCAAGATGCTGCCAACCT | GCACGATGTTCTCTGGGGTG |
| *mmu-Hk2* | GGAGAGCACGTGTGACGAC | GATGCGACAGGCCACAGCA |
| *mmu-Pkm1* | GTCTGGAGAAACAGCCAAGG | TCTTCAAACAGCAGACGGTG |
| *mmu-Pkm2* | GTCTGGAGAAACAGCCAAGG | CGGAGTTCCTCGAATAGCTG |
| *mmu-Pfkl* | CGGAGTTCCTCGAATAGCTG | CCAGCCCACTTCTTGCACCTG |
| *mmu-Ldha* | CACAAGCAGGTGGTGGACAG | AACTGCAGCTCCTTCTGGATTC |
| *mmu-lnc-Nr6a1-1* | TTGGCAGCTGCATTCCGTA | GCTTTCTCCTTCCTTTACCAGT |
| *mmu-lnc-Nr6a1-2* | TGGCTGAAGTTCCATTTCTACA | ACATCAGAACCCAAGCTGAC |
| *mmu-mtCo1* | TGCTAGCCGCAGGCATTACT | CGGGATCAAAGAAAGTTGTGTTT |
| *mmu-RNaseP* | GCCTACACTGGAGTCCGTGCTACT | CTGACCACACACGAGCTGGTAGAA |
